# Identification and functional analysis of *AvLACS* genes unveils their role in lipid homeostasis and waterlogging tolerance in kiwifruit (*Actinidia valvata* Dunn)

**DOI:** 10.3389/fpls.2025.1580003

**Published:** 2025-06-30

**Authors:** Meijuan Zhang, Kaiyu Ye, Jianyou Gao, Shibiao Liu, Ruonan Liu, Yuexing Chen, Qian Zhao, Yushan Lei, Jiewei Li, Cuixia Liu, Faming Wang

**Affiliations:** ^1^ College of Biology Pharmacy and Food Engineering, Shangluo University, Shangluo, China; ^2^ Guangxi Key Laboratory of Plant Functional Phytochemicals and Sustainable Utilization, Guangxi Institute of Botany, Guangxi Zhuang Autonomous Region and Chinese Academy of Sciences, Guilin, China; ^3^ College of Biology and Environmental Sciences, Jishou University, Xiangxi, China

**Keywords:** long-chain acyl-CoA synthetases (LACSs), *Actinidia valvata*, genome-wide characterization, expression analysis, lipid analysis, waterlogging stress

## Abstract

Long-chain acyl-CoA synthetases (LACSs) are involved in fatty acid metabolism and catabolism by converting free fatty acids to acyl-CoAs. They are essential for initiating β-oxidation of fatty acids and regulating lipid biosynthesis in plant growth and development, as well as in plant adaptation to various environmental stresses, including waterlogging stress. However, systematic identification and functional characterization of the *LACS* gene family have not been comprehensively studied in the waterlogging-tolerant kiwifruit germplasm *Actinidia valvata* Dunn. In this study, 22 *AvLACS* genes were identified within the *A. valvata* genome. The *AvLACS* genes were subsequently divided into five clusters on the basis of their phylogenetic relationships, and similar subcellular localizations, exon-intron structures, motif compositions, and protein tertiary structures were found within each cluster. Collinearity analysis identified 22 duplicated gene pairs in *A. valvata*, and these pairs have undergone purifying selection during evolution. *Cis*-acting element analysis revealed numerous hormone-responsive and stress-responsive elements in the promoter regions of the *AvLACS* genes. The expression levels of the *AvLACS* genes under waterlogging stress were determined using quantitative real-time PCR (qRT-PCR), and the results showed that the expression of the *AvLACS1.1a/b*, *AvLACS1.2a*, and *AvLACS6a/b* was significantly upregulated under waterlogging stress. Notably, *AvLACS1.1a/b* and *AvLACS1.2a* primarily facilitate short-term regulation of wax and triacylglycerol (TAG) synthesis, whereas *AvLACS6a/b* mediate TAG degradation through fatty acid β-oxidation during prolonged waterlogging. Transcriptome data revealed coordinated transcriptional regulation of TAG degradation pathway genes, which was supported by biochemical lipid profiling showing dynamic alterations in TAG content and degree of unsaturation correlated with waterlogging duration. These integrated molecular and biochemical data provide mechanistic insights highlighting distinct and coordinated roles of *AvLACS*s in lipid metabolic remodeling under waterlogging stress. These findings advance our understanding of the molecular mechanisms underlying waterlogging tolerance, and provide molecular targets and a theoretical basis for breeding waterlogging-tolerant kiwifruit and other crops.

## 1 Introduction

Lipids are structurally diverse biomolecules found in living organisms, performing essential biological functions, including energy storage, membrane formation, and signal transduction. As fundamental building blocks of lipids, fatty acids (FAs) are crucial for the formation of membrane glycerolipids, phospholipids, sphingolipids, and storage triacylglycerols (TAGs), and are indispensable for the synthesis of cell surface waxes, cutin, and suberin (Zhao et al., 2021). These molecules are vital for maintaining membrane integrity, supplying energy through metabolic pathways such as β-oxidation, and forming protective barriers against environmental stresses. In the metabolism of long-chain fatty acid-derived lipids, free FAs are activated by long-chain acyl-CoA synthetase (LACS) in a two-step enzymatic reaction to form acyl-CoA. First, free FAs react with ATP to produce an acyl-AMP intermediate, releasing pyrophosphate as a byproduct. In the second step, the enzyme-bound acyl-AMP intermediate couples with the thiol group of CoA to form acyl-CoA, releasing AMP (Groot et al., 1976; Grevengoed et al., 2014). Subsequently, the acyl-CoA produced by LACS participates in various metabolic pathways, including the synthesis of phospholipids, TAGs, cutin, cuticular wax, tryphine, and starch, as well as FA degradation via β-oxidation (Zhao et al., 2021; Li et al., 2016). Therefore, as key enzymes in FA metabolism, LACSs regulate lipid biosynthesis and energy homeostasis, playing critical roles in plant growth and adaptation to environmental stresses.

LACSs are a widespread family of enzymes crucial for lipid metabolism and are highly conserved across multicellular organisms. Since the identification of nine *LACS* family members in Arabidopsis (Shockey et al., 2002), numerous *LACS* members have been characterized in various species, including economically crops such as rice (*Oryza sativa* L.) (Kitajima-Koga et al., 2020), maize (*Zea mays* L.) (Wang et al., 2023; Yan et al., 2024) and wheat (*Triticum aestivum* L.) (Liu et al., 2022), oilseed crops such as soybean [*Glycine max* (L.) Merr.] (Wang et al., 2022), cotton (*Gossypium* spp.) (Zhong et al., 2023), rapeseed (*Brassica napus* L.) (Xiao et al., 2019), pecan [*Carya illinoinensis* (Wangenh.) K. Koch] (Ma et al., 2023), sunflower (*Helianthus annuus* L.) (Aznar-Moreno et al., 2014) and African oil palm (*Elaeis guineensis* Jacq.) (Wang et al., 2021), fruit crops such as apple [*Malus domestica* (Suckow) Borkh.] (Zhang et al., 2018) and ‘Dangshansuli’ pear (*Pyrus bretschneideri* rehd.) (Wang et al., 2025) and vegetable crop like tomato (*Solanum lycopersicum* L.) (Wu et al., 2024). In plants, although there are differences in the substrate specificity, subcellular distribution, and expression patterns of different *LACS* members, all LACS members possess a highly conserved AMP-binding domain, which is essential for the activation of long-chain fatty acids (LCFAs) to their respective long-chain-acyl-CoA thioesters (Zhao et al., 2021; Lewandowska et al., 2020). Additionally, a conserved acyl CoA synthetase (ACS) signature motif plays important roles in this activation process, facilitating the conversion of LCFAs to acyl-CoA thioesters (Ma et al., 2023; Zhou et al., 2024).

LACS enzymes are primarily localized in the endoplasmic reticulum (ER), peroxisome, plastid, and plasma membrane (PM), and the subcellular localization of LACS determines the subsequent fatty acid metabolic pathway it participates in. For example, LACSs localized to the plastid envelope, such as AtLACS9 in Arabidopsis, are involved in the activation of *de novo* synthesized LCFAs and facilitate lipid trafficking between plastids and the ER (Kitajima-Koga et al., 2020; Jessen et al., 2014). Peroxisome-localized LACSs, such as AtLACS6 and AtLACS7, play key roles in the degradation of TAGs to provide energy and maintain lipid homeostasis via FA β-oxidation (Yu et al., 2014; Wang et al., 2019). The majority of AtLACSs, including AtLACS1, AtLACS2, AtLACS4 and AtLACS8, are located in the ER, where FAs are metabolized to synthesize membrane lipids, cuticular lipids, and TAG (Zhao et al., 2021; Shockey et al., 2002; Kunst and Samuels, 2003; Fich et al., 2016). These generated lipids are also active components of plant adaptations to biotic and abiotic stresses. Membrane lipid remodeling is a key strategy for plants to adapt to environmental stresses (Zhang et al., 2016; Wang et al., 2014; Henschel et al., 2024). Additionally, the sequestration of acyl groups into TAGs stored in lipid droplets helps maintain cellular homeostasis under stress conditions (Wang et al., 2019; Yu et al., 2021). The cuticle on the cell surface, which is composed of cuticular wax and cutin, serves as a critical barrier against environmental stresses, including water stress (Bernard and Joubès, 2013). Thus, these LACS-mediated FA metabolic processes and their lipid products are crucial for plant growth and development, and are actively induced under various environmental stresses.

LACSs are involved in plant responses to various biotic factors, such as pathogen attacks, as well as abiotic stresses including drought, salinity, and hypoxia. In Arabidopsis, a reduction in wax and cutin, which function as a surface barrier against pathogens and water deprivation, was observed in the *lacs1* and *lacs2* mutant plants. As a result, the mutants exhibited increased susceptibility to the bacterial pathogen *Pseudomonas syringae* (Bernard and Joubès, 2013; Tang et al., 2007) as well as to drought stress (Weng et al., 2010). The expression of *MdLACS1* was upregulated under drought stress, salt stress, and abscisic acid (ABA) treatment in apple (Zhang et al., 2018). Furthermore, *MdLACS2* and *MdLACS4* also contribute to enhanced drought and salt stress resistance in transgenic plants (Zhang et al., 2020a, Zhang et al., 2020b). Similarly, *GmLACS2–3* in soybean (*Glycine max* L.), *PoLACS4* in tree peony (*Paeonia ostii* ‘Feng Dan Bai’), and *CiLACS9* and *CiLACS9–1* in pecan (*Carya illinoinensis* L.) were highly expressed under drought and salt stresses (Ma et al., 2023; Zhang et al., 2022; Ayaz et al., 2021). Temperature fluctuations also affect the expression of *LACS*. For example, the expression of *GmLACS9, GmLACS15*, and *GmLACS17* in soybean was signifiantly upregulated under low temperature (Wang et al., 2022), whereas *ZmLACS9* was significantly induced by heat stress (Wang et al., 2023). Furthermore, hypoxia stress, induced by waterlogging, increased the expression of *LACS6* in cucumber (*Cucumis sativus* L.) (Kęska et al., 2021) and four *AvLACSs* in kiwifruit (*Actinidia valvata* Dunn) (Li et al., 2022), suggesting that LACS plays a vital role in the plant response to waterlogging stress. Additionally, the response mechanism of AtLACS2 to hypoxia stress has been thoroughly investigated. AtLACS2 in Arabidopsis contributes to submergence tolerance by modulating the translocation of the ETHYLENE-RESPONSE FACTOR (ERF-VII) transcription factor from the membrane to the nucleus (Zhou et al., 2020) and by modulating cuticle permeability in plant cells (Xie et al., 2020).

Kiwifruit (*Actinidia* spp.) is a significant perennial fruit crop in the genus Actinidia and comprises more than 50 species (Huang, 2016). Kiwifruit is highly valued because of its high vitamin C content and essential minerals, which are beneficial for human health. However, most kiwifruit cultivars are highly sensitive to waterlogging stress due to their fleshy roots, resulting in reduced fruit yield under waterlogged conditions. Previous research has focused on screening for waterlogging-tolerant kiwifruit plants, and *A. valvata* has been identified as a highly waterlogging-tolerant kiwifruit variety and is now widely used as a rootstock in kiwifruit production (Li et al., 2021; Bai et al., 2022). To investigate the adaptive strategies employed by *A. valvata* under waterlogging stress, Li et al. (2022) conducted a transcriptome analysis on the roots of KR5 (*A. valvata*, a tolerant genotype) after 0, 12, 24, and 72 h of waterlogging stress, and the results showed that four differentially expressed genes (DEGs) encoding LACS were significantly upregulated under waterlogging stress. However, the systematic identification and characterization of the kiwifruit *LACS* gene family remain unstudied. In this study, genome-wide identification and investigation of the *LACS* gene family were conducted in *A. valvata*. The chromosomal locations, phylogenetic relationships, conserved domains, gene structures, and *cis*-acting elements were analyzed, along with gene synteny analysis and the expression patterns of *AvLACSs* at different fruit development stages and under salt stress. Additionally, the transcriptional level of *AvLACSs* under waterlogging stress was quantified by qRT-PCR analysis, and the functional mechanism of the upregulated genes was analyzed via the transcriptome analysis. Further lipid profiling was performed to validate the regulatory role of AvLACSs in wax synthesis and TAG metabolism under waterlogging stress. Our findings provide a scientific foundation for the functional validation of *LACS* genes in waterlogging stress adaptation, and offer valuable insights into breeding waterlogging-tolerant kiwifruit and variety identification.

## 2 Materials and methods

### 2.1 Identification of *LACS* Genes in *A. valvata*

We sequenced the entire genome of the *A. valvata* (BGI Inc., Shenzhen, China) and the genome sequencing data have been deposited at Sequence Read Archive database in NCBI under accession PRJNA1169670. A systematic annotation of genomic features, including protein-coding genes, was performed (unpublished), and the corresponding protein sequences were subsequently obtained. To identify the *LACS* gene family members from the *A. valvata* genome, we downloaded the HMM file of the AMP-binding domain (PF00501) using the Pfam database (https://pfam.xfam.org/) (accessed on 29 March 2024) (Mistry et al., 2020). The AMP domain was used to search the *A. valvata* protein database with HMMER 3.0 (https://www.ebi.ac.uk/Tools/hmmer/) (accessed on 29 March 2024) (Potter et al., 2018) with a threshold of E-value ≤ 1e^−5^ and other parameters set to defaults. Then, nine LACS protein sequences of *A. thaliana* were downloaded from The Arabidopsis Information Resource (TAIR, https://www.arabidopsis.org/) (accessed on 29 March 2024) (Lamesch et al., 2011) and used for the BLASTp analysis with the kiwifruit protein sequences with an E-value threshold of ≤1e^−5^. The merged results of these two methods were used to conduct a phylogenetic analysis with the 9 AtLACS proteins, 22 putative AvLACS proteins were finally identified. The putative AvLACS protein sequences were submitted to the NCBI-CDD website (https://www.ncbi.nlm.nih.gov/cdd) (accessed on 8 May 2024) and the SMART databases (http://smart.embl-heidelberg.de/) (accessed on 8 May 2024) to further confirm the existence of the AMP binding domain. These AvLACS members were named according to their affinities to AtLACS from *A. thaliana*.

### 2.2 Chromosomal location and physicochemical properties of the *AvLACS* genes

The chromosomal location of the *AvLACSs* was achieved from a gff3 file of the genome and mapped to different chromosomes using MG2C v2.1 online software (http://mg2c.iask.in/mg2c_v2.1/) (accessed on 26 May 2024) (Chao et al., 2021). The physicochemical properties of the AvLACS proteins, namely, the amino acid (A.A) length, molecular weight (M.W), isoelectric point (pI), instability index, the aliphatic index and grand average of hydropathicity (GRAVY) were evaluated by using the ExPASy website (https://www.expasy.org/) (accessed on 8 May 2024). Meanwhile, the subcellular localization of the AvLACS proteins was predicted using the PredictProtein web server(https://predictprotein.org/) (accessed on 9 May 2024) (Bernhofer et al., 2021).

### 2.3 Phylogenetic analysis, conserved motifs and gene structure analysis of the *AvLACS* genes

Multiple sequence alignment of 22 AvLACS proteins and 9 AtLACS proteins was performed using the Clustal W method in DNAMAN 8.0 software (Lynnon Biosoft, Vaudreuil, QC, Canada) with default parameters. To construct the phylogenetic tree, the *LACS* members from *A. thaliana*, *A. valvata*, *Z. mays* (Wang et al., 2023), and *O. sativa* (Ma et al., 2023) were renamed in accordance with their affinities to Arabidopsis (**Supplementary Table 1**). The evolutionary relationship of LACS family members was established by constructing a phylogenetic tree using MEGA 7.0 software (Mega Limited, Auckland, New Zealand) with the neighbor-joining (NJ) method following the default settings. A bootstrap analysis with 1000 replicates was performed, and the resulting tree were visualized via the online web tool iTOL (https://itol.embl.de/) (accessed on 16 May 2024) (Letunic and Bork, 2021). Gene structure information regarding the intron-exon distribution of *AvLACS* genes was retrieved from the General Feature Format (GFF) file. The conserved motifs of AvLACS protein sequences were predicted using the MEME (MEME 5.5.4) online tool (https://memesuite.org/meme/tools/meme) (accessed on 8 May 2024) (Bailey et al., 2015) and the maximum number of motifs was 10. The phylogenetic tree, gene structures and conserved motifs of AvLACS were visualized using the Gene Structure View (Advanced) within TBtools II software (Chen et al., 2023).

### 2.4 Conserved domains, secondary structure and 3D modeling of AvLACS proteins

The conserved domains in all AvLACS proteins were analyzed using the SMART database, and the domain structures were plotted using IBS 1.0.3 software (Liu et al., 2015). The secondary structures of the AvLACS proteins were predicted using SOPMA (https://npsa-prabi.ibcp.fr/cgi-bin/npsa_automat.pl?page=npsa_sopma.html) (accessed on 13 May 2024) (Geourjon and Deléage, 1995). Furthermore, we constructed 3D models of AvLACS proteins based on protein homology modeling using the online tool Phyre2 (http://www.sbg.bio.ic.ac.uk/phyre2/html/page.cgi?id=index) (accessed on 12 May 2024) (Kelley et al., 2015) with default parameters.

### 2.5 Gene duplication, collinearity analysis and Ka/Ks values calculation of *AvLACS* genes

Gene replication of *AvLACS* genes were identified by TBtools using the MCScanX toolkit package with default parameters (Chen et al., 2023). The collinearity of the *LACS* family members within *A. valvata* genome as well as between *A. valvata* and *A. thaliana* and *A. chinensis*, was determined using TBtools software (Chen et al., 2023). The Ka (non-synonymous) and Ks (synonymous) substitution rates and the Ka/Ks ratio of the duplicated *AvLACS* genes were calculated using TBtools software (Chen et al., 2023). Ka/Ks < 1 suggests the presence of purifying selection, whereas Ka/Ks > 1 implies positive selection and Ka/Ks = 1 is characteristic of neutral selection (Zhang, 2022). The divergence time (T, MYA; million years ago) was calculated by the following formula; T = Ks/r, (r = 6.78 × 10^−9^) (Luo et al., 2023; Tu et al., 2023).

### 2.6 *Cis*-regulatory element analysis of *AvLACS* genes

To predict the putative *cis*-regulatory elements in promoter regions of *AvLACS* genes, the 2000 bp upstream sequences of all the *AvLACS* genes were extracted from the genomic DNA sequences. The promoter sequences of each gene were submitted to PlantCARE (https://bioinformatics.psb.ugent.be/webtools/plantcare/html/) (accessed on 23 May 2024) (Lescot et al., 2002), and the distribution and the numbers of *cis*-regulatory elements were visualized in TBtools (Chen et al., 2023).

### 2.7 Gene expression analysis of *AvLACS* genes based on RNA-seq data

To investigate the expression pattern of *AvLACS* genes during plant development and under abiotic stresses, the publicly available *A. valvata* RNA-seq datasets deposited in the NCBI Sequence Read Archive (SRA) database were searched and downloaded (PRJNA984935 and PRJNA726156) (accessed on 1 November 2023). For plant development, the fruit flesh excluding seeds were collected at four different stages of fruit development (Green stage, Breaker stage, Color change stage and Mature ripe stage) from *A. valvata* (Bhargava et al., 2023). For salt stress, plants of *A. valvata* were subjected to 0.4% NaCl per net weight of the growing medium in the pot, and the roots of *A. valvata* were collected at 0 h (control), 12 h, 24 h and 72 h after the salt treatment (Abid et al., 2022). The RNA-seq data were converted intoFASTQ format using the Convert SRA to Fastq Files within TBtools II software (Chen et al., 2023). Subsequently, the transcriptional abundance of all transcripts was quantified using Kallisto Super GUI Wrapper of TBtools with default parameters through uploading the FASTQ files. The resulting expression values were normalized as Transcripts Per Million (TPM) and the normalized expression data of *AvLACS* genes were extracted using the Table Row Extractor and Filter tools within TBtools (Chen et al., 2023; Qiao et al., 2025). The Heatmap of TBtools was used to normalize data and draw heatmaps (Chen et al., 2023).

### 2.8 Plant materials and waterlogging treatments

Two-year-old *A. valvata* seedlings were cultivated in the greenhouse at Guangxi Key Laboratory of Plant Functional Phytochemicals and Sustainable Utilization and grown under normal conditions for three months. For the waterlogging experiment, each three potted seedings were placed in a plastic container (61 × 48 × 36 cm) filled with tap water. The seedlings were submerged to a final depth range of 3~5 cm beneath the water surface for 7 days under normal light/dark conditions (Zhang et al., 2024). Fresh root samples were collected at 0 h, 6 h, 24 h (1 d), 120 h and 7 d after the waterlogging treatment and immediately frozen in liquid nitrogen for further analyses. For each sample, a set of three replicates was established, with each replicate comprising three individual seedlings.

### 2.9 RNA extraction and qRT-PCR analysis

The total RNA of the root samples was extracted using the RNAprep Pure Plant Kit (TIANGEN, Beijing, China) according to the manufacturer’s instruction. The RNA integrity was checked using 1% agarose gel electrophoresis, and the RNA purity and concentration were measured using a spectrophotometer (UV-2550; Shimadzu, Co., Kyoto, Japan). The FastKing RT Kit With gDNase (TIANGEN, Beijing, China) was used to reverse the RNA into cDNA. Real-time PCRs were performed using the SuperReal PreMix Plus (SYBR Green) (TIANGEN, Beijing, China) following the manufacturer’s instructions. The relative expression levels were calculated using the 2^−ΔΔCT^ method (Livak and Schmittgen, 2001) and three duplicates were performed. The primer pairs used for the qRT-PCR are provided in **Supplementary Table 2**.

### 2.10 Transcriptomic datasets analysis

The root samples were sent to Biomarker Technologies Co., Ltd. (Beijing, China) and performed transcriptome sequencing with three biological replicates. Total RNA was extracted from these samples using the RNAprep Pure Plant Kit (Tiangen, Beijing, China). RNA concentration and purity was measured using NanoDrop 2000 (Thermo Fisher Scientific, Wilmington, DE). RNA integrity was assessed using the RNA Nano 6000 Assay Kit of the Agilent Bioanalyzer 2100 system (Agilent Technologies, CA, USA). A total amount of 1 μg RNA per sample was used for library preparation with the Hieff NGS Ultima Dual-mode mRNA Library Prep Kit for Illumina (Yeasen Biotechnology, Co., Ltd. Shanghai, China) following the manufacturer’s instructions. The libraries were sequenced on an Illumina NovaSeq platform to generate 150 bp paired-end reads. Gene annotation was performed by comparing the DEGs with COG (Cluster of Orthologous Group of Proteins), GO (Gene Ontology), KEGG (Kyoto Encyclopedia of Genes) and Genomes, Swiss-prot, and NR (Non-redundant) databases. KEGG pathway enrichment analysis was conducted on the samples, and the DEGs were classified into regulatory pathways based on the annotation results. Based on RNA-seq data of *A. valvata* (Unpublished), the FPKM values mapped reads were used to calculate the expression levels of genes related to FAs metabolism under normal and waterlogging conditions, and the results were visualized by heat maps using TBtools (Chen et al., 2023).

### 2.11 Lipid analysis

For lipid analysis, the three biological samples were sent to BioMarker Technologies (Beijing, China). Root samples (100 mg) of 0 d, 1 d and 7 d after the waterlogging treatment were dissolved with 1.2 ml of 70% methanol, after vortexed for six times and then the samples were placed in the refrigerator at 4 °C overnight. After centrifugation, the supernatant was filtered and stored in an injection bottle for UPLC-MS/MS analysis using an Agilent SB-C18 column with a specific elution gradient of 0.1% formic acid aqueous solution and 0.1% formic acid acetonitrile. LC-MS/MS was performed on a triple quadrupole-linear ion trap mass spectrometer (QTRAP)in both positive and negative modes, with specific source parameters and mass calibration. The double bond index (DBI) was calculated from the mol % values derived from the LC-MS/MS data, according to the formula: DBI = [∑(number of double bonds × mol % of fatty acid)]/100 (Falcone et al., 2004).

### 2.12 Statistical analysis

Statistical analysis was performed using one-way ANOVA and Tukey’s test in SPSS (ANCOVA; SPSS26, SPSS Inc., Chicago, IL, USA), and * *P* < 0.05 and ** *P* < 0.01 indicated that the differences were significant and extremely significant, respectively. The data are presented as the mean ± standard error (± SE) of three biological replicates, with each treatment was repeated three times.

## 3 Results

### 3.1 Identification of *LACS* Gene Family Members in *A. valvata*

**Figure 1:**
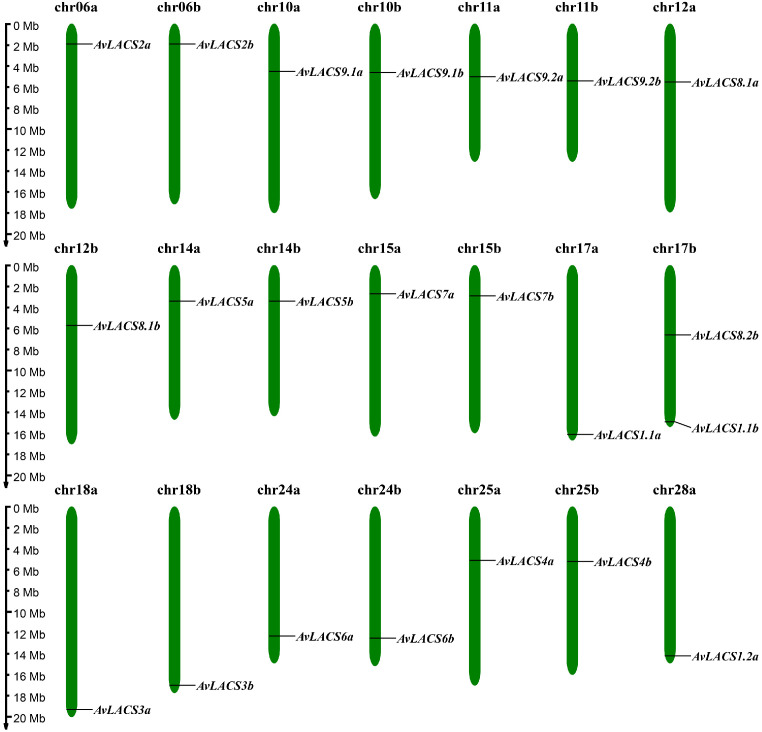
Chromosomal distribution of the *AvLACSs* in *A. valvata*.

To identify *LACS* gene family members in *A. valvata*, we performed BLASTP searches and Hidden Markov Model (HMM), and the results were merged and filtered through phylogenetic analysis. NCBI-CDD and SMART domain searches were employed to verify the presence of AMP-binding domains in putative *AvLACS* candidate genes. Finally, 22 *AvLACS* family members were identified from the genome of *A. valvata*, and they were renamed according to their similarity to Arabidopsis (**Supplementary Figure 1**). All of the identified *AvLACS* genes were found to be located on 21 chromosomes, with 11 *LACS* genes on each subgenome a and subgenome b (**Figure 1**; **Supplementary Table 3**). The CDS lengths of the *AvLACS* genes ranged from 1437 to 2205 bp, with protein lengths varying between 478 and 734 amino acids (**Table 1**). Their molecular weights ranged from 54.26 to 80.61 kDa, and their pI values ranged from 5.79 to 8.53. The instability index of these AvLACS proteins ranged from 22.83 to 40.35, and their aliphatic index ranged from 83.81 to 96.85, and their grand average of hydropathicity (GRAVY) ranged from -0.295 to 0.09. Subcellular localization predictions indicated that 11 AvLACS proteins were located in the endoplasmic reticulum, 4 were located in the peroxisome, and 7 were located in the chloroplast (**Table 1**).

**Table 1:** Detailed information on the *LACS* family members in *A. valvata*.

Gene name	Gene ID	Chromosome	CDS length (bp)	Number of amino acids (aa)	Molecular weight (kDa)	pI	Instability index	Aliphatic index	Grand average of hydropathicity	Subcellular localiaztion
*AvLACS1.1a*	AVa17g01181.1	chr17a	1437	478	54.26	6.02	40.35	92.76	-0.295	ER^1^
*AvLACS1.1b*	AVb17g01151.1	chr17b	1629	542	61.16	6.07	39.10	93.67	-0.157	ER
*AvLACS1.2a*	AVa28g01307.1	chr28a	1926	641	72.36	6.70	36.24	86.05	-0.261	ER
*AvLACS2a*	AVa06g00244.1	chr06a	1980	659	73.70	5.79	35.89	86.56	-0.195	ER
*AvLACS2b*	AVb06g00239.1	chr06b	2148	715	80.61	6.07	38.96	89.16	-0.134	ER
*AvLACS3a*	AVa18g01402.1	chr18a	1983	660	73.33	6.13	32.94	93.94	-0.069	ER
*AvLACS3b*	AVb18g01322.1	chr18b	1983	660	73.44	6.13	33.79	95.83	-0.060	ER
*AvLACS4a*	AVa25g00543.1	chr25a	1977	658	73.48	6.66	35.46	88.98	-0.118	ER
*AvLACS4b*	AVb25g00540.1	chr25b	1977	658	73.59	6.25	35.75	89.15	-0.113	ER
*AvLACS5a*	AVa14g00350.1	chr14a	1977	658	73.51	6.14	36.60	88.95	-0.122	ER
*AvLACS5b*	AVb14g00362.1	chr14b	1977	658	73.57	6.05	39.15	88.95	-0.130	ER
*AvLACS6a*	AVa24g01059.1	chr24a	2121	706	77.63	7.51	27.28	85.61	-0.116	Peroxisome
*AvLACS6b*	AVb24g01058.1	chr24b	2121	706	77.62	7.82	29.34	83.81	-0.136	Peroxisome
*AvLACS7a*	AVa15g00302.1	chr15a	2076	691	76.53	7.24	36.18	89.45	-0.114	Peroxisome
*AvLACS7b*	AVb15g00324.1	chr15b	2076	691	76.80	6.79	36.50	89.31	-0.122	Peroxisome
*AvLACS8.1a*	AVa12g00471.1	chr12a	2196	731	79.75	8.43	26.69	92.42	-0.012	Chloroplast
*AvLACS8.1b*	AVb12g00450.1	chr12b	2205	734	80.19	8.51	27.09	92.98	-0.006	Chloroplast
*AvLACS8.2b*	AVb17g00537.1	chr17b	2145	714	78.23	8.53	22.83	95.17	0.036	Chloroplast
*AvLACS9.1a*	AVa10g00479.1	chr10a	2166	721	79.29	8.09	36.08	96.42	-0.005	Chloroplast
*AvLACS9.1b*	AVb10g00463.1	chr10b	2094	697	76.76	8.26	34.29	93.44	-0.058	Chloroplast
*AvLACS9.2a*	AVa11g00469.1	chr11a	1776	591	64.39	6.48	35.57	96.85	0.09	Chloroplast
*AvLACS9.2b*	AVb11g00477.1	chr11b	1575	524	57.65	5.92	31.88	95.44	0.02	Chloroplast

^1^ Note: ER, endoplasmic reticulum.

### 3.2 Phylogenetic analysis and characterization of conserved domains of LACSs in *A. valvata*

**Figure 2:**
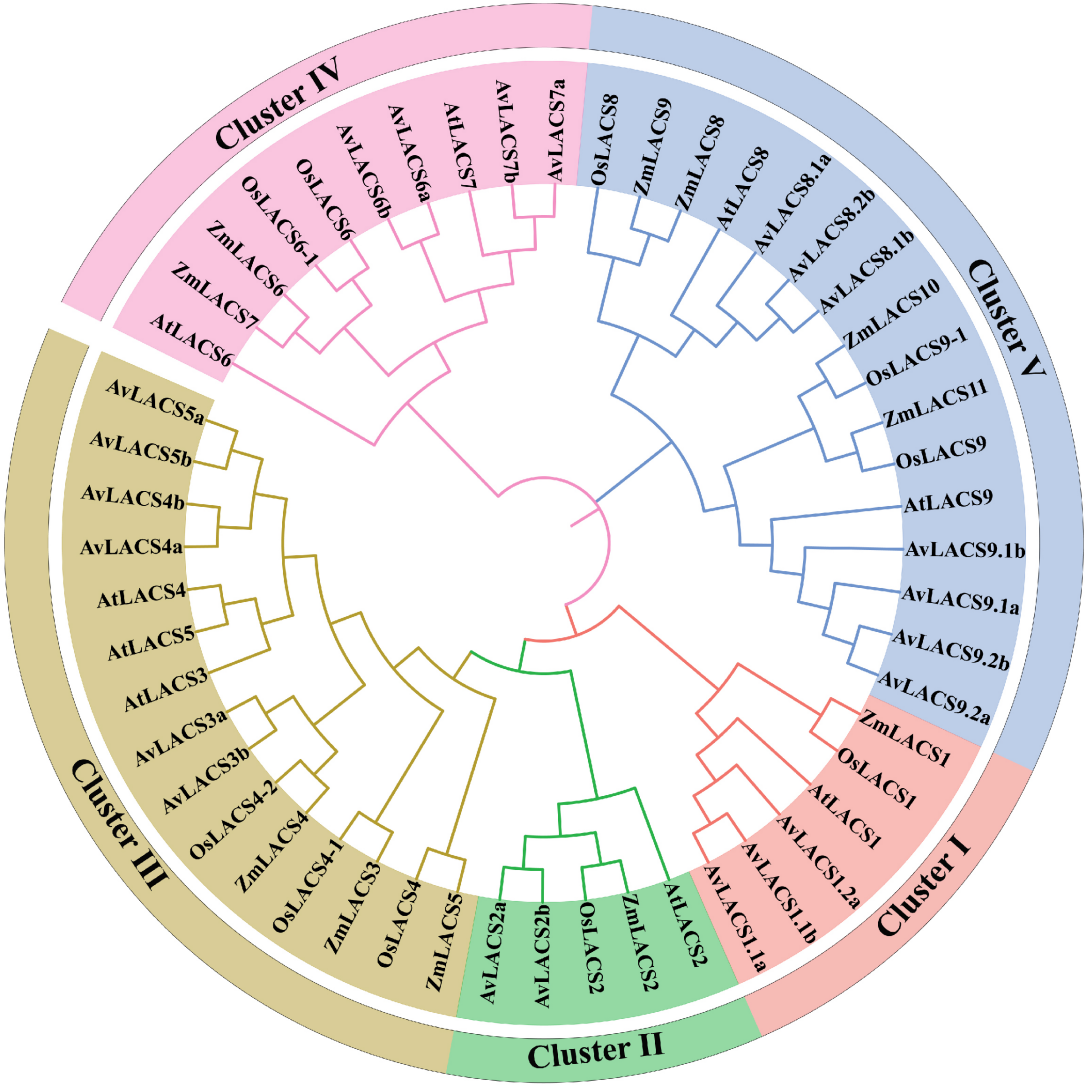
Phylogenetic analysis of the LACS proteins from *A. valvata* (Av), *A. thaliana* (At), *O. sativa* (Os), and *Z. mays* (Zm). The protein sequences were aligned with the Clustal W program using MEGA 7.0 and the phylogenetic tree was constructed using the neighbor-joining method with 1000 bootstrap replicates.

To explore the phylogenetic relationships and functional diversity of the AvLACSs, the LACS protein sequences from *A. valvata*, *A. thaliana*, *Z. mays*, and *O. sativa* were subjected to multiple sequence alignment, and a phylogenetic tree of these LACS proteins was constructed. The LACSs were divided into five clusters (**Figure 2**). Cluster I included *AvLACS1.1a/b* and *AvLACS1.2b*, cluster II contained the fewest genes, which were *AvLACS2a/b*; cluster III consisted of *AvLACS3a/b*, *AvLACS4a/b*, and *AvLACS5a/b*; cluster IV consisted of *AvLACS6a/b*, and *AvLACS7a/b*; and cluster V contained the highest number of genes, which were *AvLACS8.1a/b*, *AvLACS8.2b*, *AvLACS9.1a/b*, and *AvLACS9.2a/b*. The same *AvLACS* genes located on different subgenomes were phylogenetically closer, whereas *LACSs* from monocots on the evolutionary branch were relatively more distant from *AvLACSs*. The subcellular localization of LACS proteins clustered together on the phylogenetic tree is also consistent.

The conserved domains of the AvLACSs were analyzed using the SMART database and visualized with IBS software (version 1.0.3). As shown in **Supplementary Figure 2**, most AvLACSs contained an AMP-binding domain (PF00501). Compared with LACSs from other clusters, the AvLACSs from cluster II, namely, AvLACS2a/b, possessed an additional AMP-binding C domain. Similar domain positions and distributions were found within the same cluster.

### 3.3 protein sequence alignment and protein structure analysis of AvLACSs

To identify the conserved sequences in the AvLACSs, multiple sequence alignment of proteins was performed using DNAMAN software (**Supplementary Figure 3**). According to the alignment, each AvLACS protein contained two conserved motif domains (AMP-binding domain signature and ACS signature motif). In addition, the amino acid sequences of the conserved motif domain in AvLACS located within the same cluster were highly similar.

**Figure 3:**
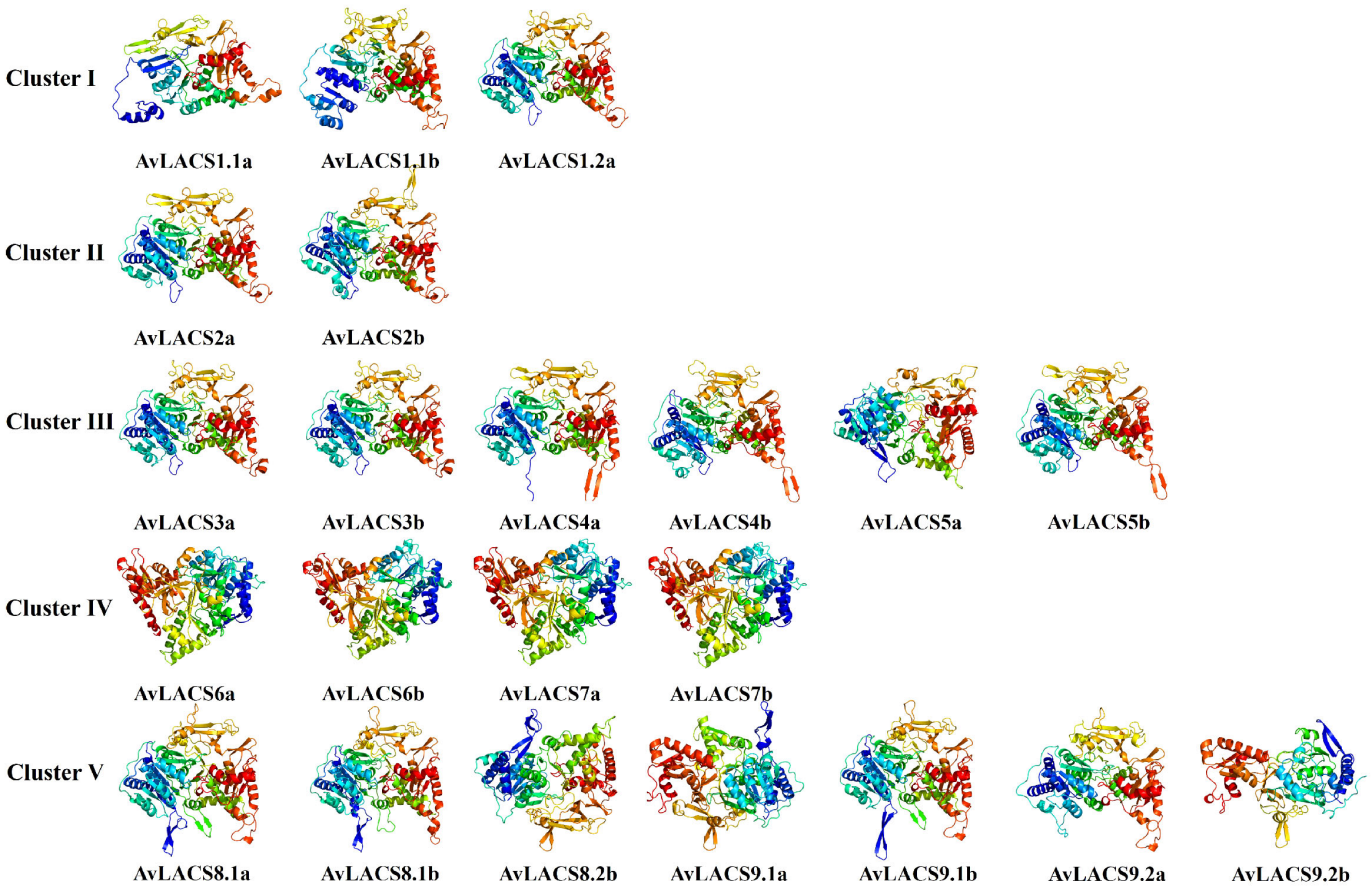
Three-dimensional (3D) structure of AvLACS proteins. The 3D models were constructed using the online Phyre2 server in default mode.

To further understand the AvLACS protein structure, the secondary and tertiary structures of AvLACS were predicted. The protein secondary structure of the AvLACS family members was predominantly composed of α-helices (35.70%-42.23%) and random coils (33.89%-38.00%), followed by extended strands (16.21%-20.64%), whereas β-turns (6.11%-8.64%) accounted for the smallest proportion (**Supplementary Figure 4**; **Supplementary Table 4**). Additionally, three-dimensional models of all AvLACSs proteins were built. As shown in **Figure 3**, similar 3D structures were found in the AvLACSs from the same cluster, and the composition and position of the secondary structures of the AvLACS proteins could be clearly observed.

### 3.4 Gene structure and conserved motif analysis of *AvLACS*

**Figure 4:**
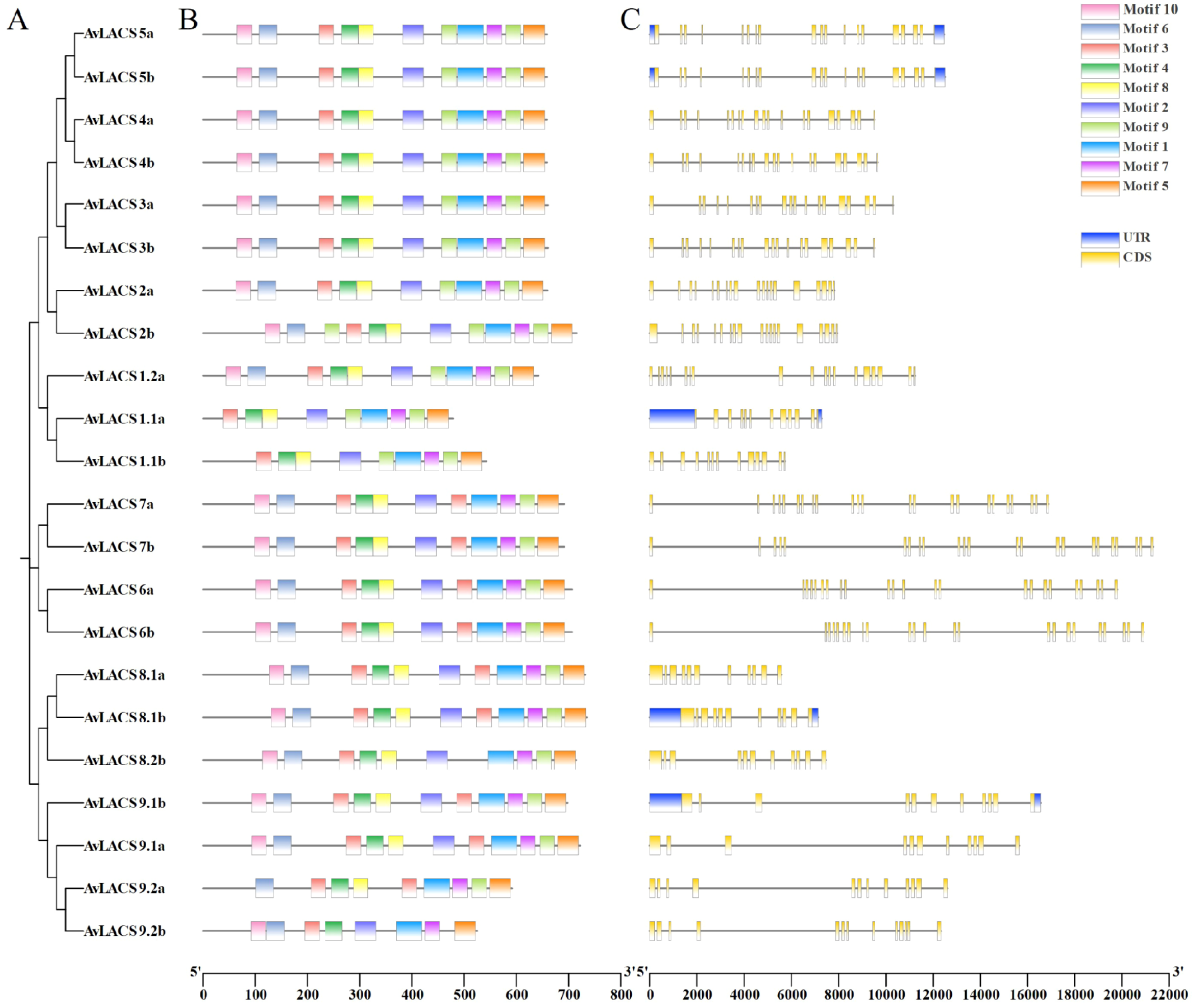
The phylogenetic tree, conserved motif, and gene structure of the *AvLACS* genes. **(A)** Phylogenetic tree of AvLACS proteins. **(B)** Conserved motif distribution of AvLACS proteins. **(C)** Exon-intron structure of *AvLACS* genes.

To further investigate the evolutionary conservation of the *AvLACS* family genes, the conserved motifs and exon-intron organization of the *AvLACS* gene structure were analyzed (**Figure 4**). We predicted 10 conserved motifs in all the AvLACS proteins using the MEME software (**Figure 4B**; **Supplementary Figure 5**). The results revealed that a similar motif distribution was observed within each cluster. For instance, the AvLACS proteins within cluster III presented all 10 motifs, all of which were arranged in the same sequence. Interestingly, they contained two motif 9. Cluster IV also contained all 10 motifs and all motifs were arranged in the same order. However, unlike cluster III, all the AvLACS from cluster IV contained two motif 3. Gene structure analysis revealed that the *AvLACS* genes within the same cluster presented similar exon-intron gene structures (**Figure 4C**; **Supplementary Figure 6**). For example, the cluster II and III members all contained 19 exons and 18 introns, while the cluster IV members all contained 23 exons and 22 introns. Most members of Cluster V had 11 exons and 10 introns, with the exception of *AvLACS9.2a/b*.

### 3.5 Gene duplication and collinearity analysis of *AvLACS* genes

**Figure 5:**
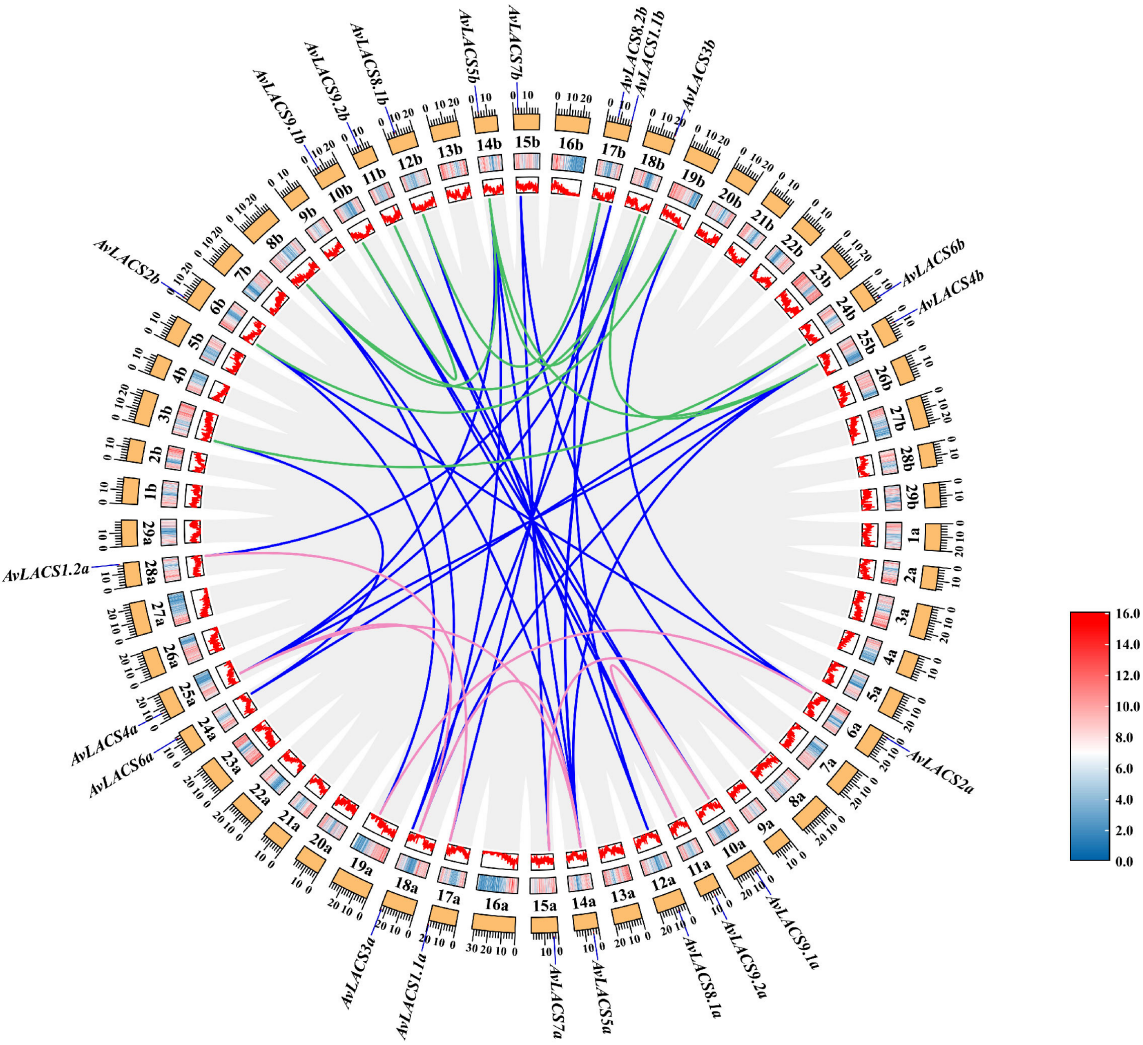
A collinearity analysis of *AvLACSs*. The grey lines indicate all duplicate genes, while the blue lines indicate the duplicated *LACS* gene pairs between *A*. *valvata* subgenome a and subgenome b. The pink lines in the circle indicate the duplicated *LACS* gene pairs within the subgenome a, and the green lines indicate the duplicated *LACS* gene pairs within the subgenome b. The heatmap and line graph indicate gene density.

To elucidate the synteny relationships among homologous *LACS* genes and infer gene duplication events, we conducted a collinearity analysis by using MCScanX. A total of 22 duplicated gene pairs were found in the *A. valvata* genome (**Figure 5**; **Supplementary Table 5**). The kiwifruit *A. valvata* is a tetraploid, and its genome can be divided into subgenome a and subgenome b. Each subgenome a or subgenome b has 3 duplicated gene pairs. However, there were 16 duplicated gene pairs between subgenome a and subgenome b. Then, we analyzed the selection pressure of replicated gene pairs by calculating the nonsynonymous (Ka) and synonymous (Ks) substitution rates. In *A. valvata*, all duplicated *AvLACS* gene pairs showed a Ka/Ks ratio of less than 1, indicating that they have undergone purifying selection during evolution (**Supplementary Table 5**). Additionally, the results showed that the gene duplication events occurred approximately 4.37 to 67.68 million years ago (MYA). In subgenome a, the gene duplication events occurred at 24.27, 25.90 and 67.12 MYA, while in subgenome b, the gene duplication events took place at 19.35, 25.98 and 67.45 MYA.

To further elucidate the orthologous relationships of the *AvLACS* gene family, a multicollinearity analysis was conducted between *A. valvata*, *A. thaliana* and *Actinidia chinensis* ‘Hongyang’ (**Supplementary Figure 7**). Within *A. valvata*, there were 16 *AvLACS* gene pairs between subgenome a and subgenome b (**Supplementary Figure 7A**). The result showed that 14 and 15 gene pairs of *LACS* collinearity were found between *A. chinensis* ‘Hongyang’ and *A. valvata* subgenome a and subgenome b, respectively (**Supplementary Figure 7B**). In addition, there were each 14 gene pairs of *LACS* collinearity between *A. thaliana* and the *A. valvata* subgenome a or subgenome b (**Supplementary Figure 7C**).

### 3.6 *Cis*-acting elements analysis in the promoter regions of the *AvLACS* genes

**Figure 6:**
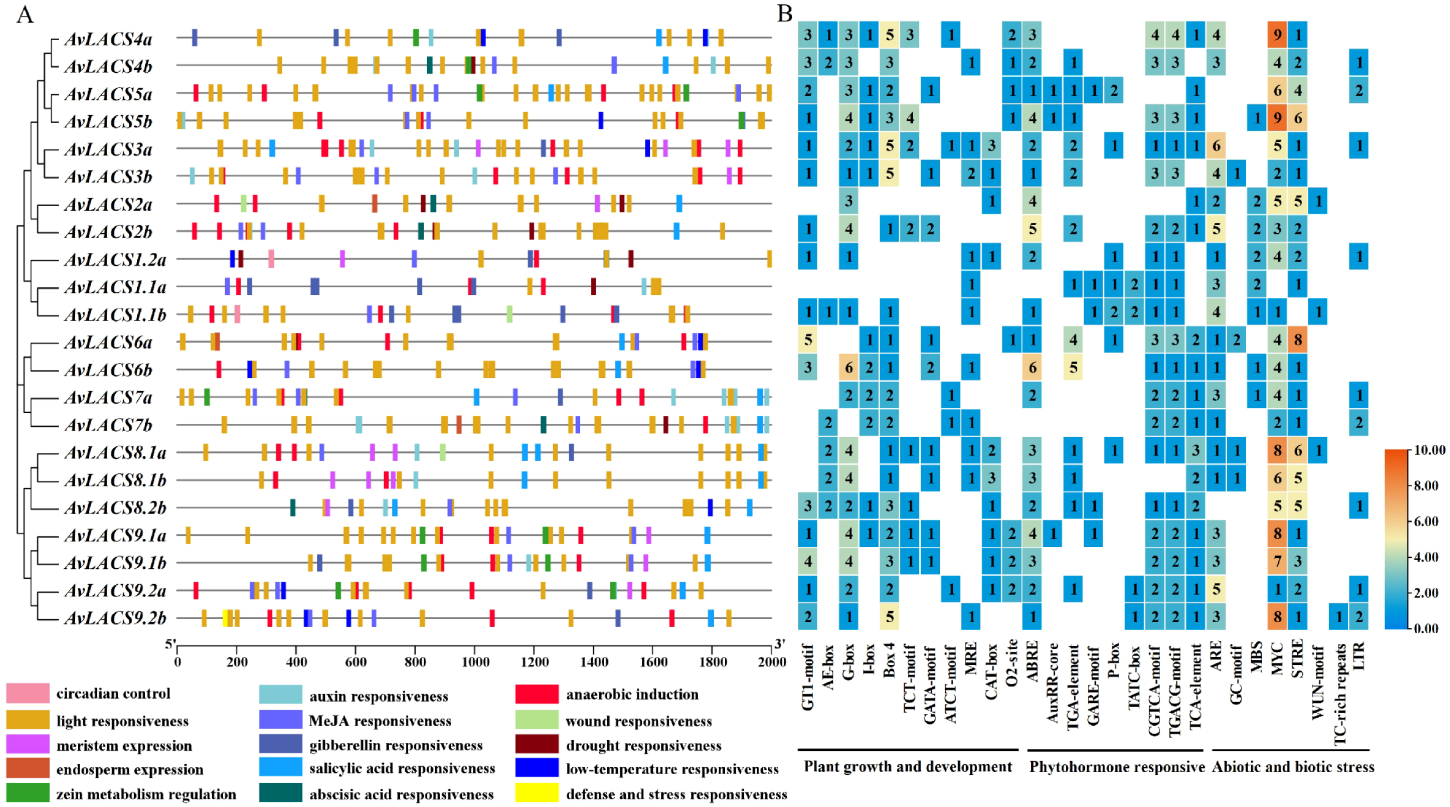
The *cis*-acting elements in the *AvLACS* gene promoter. **(A)** *Cis*-acting elements distribution in the promoter regions, with each colored rectangle representing a distinct type of *cis*-element; **(B)** Statistics on the number of *cis*-acting elements associated with plant growth and development and phytohormone and stress responses in the promoter region of *AvLACS* genes.

To predict the potential molecular functions of the *AvLACS* genes, the *cis*-acting elements located in the upstream 2,000 bp promoter regions of all the *AvLACS* genes were analyzed using PlantCARE. The distribution and numbers of these *cis*-acting elements on the promoters of the *AvLACS* genes are shown in **Figure 6**. All of the *AvLACS* gene promoter regions contained numerous light response *cis*-elements (**Figure 6A**), such as GT1-motif, AE-box, and G-box (**Figure 6B**). The phytohormone-responsive *cis*-acting elements, especially the methyl jasmonate (MeJA) and abscisic acid (ABA) responsive elements, are widely distributed. The phytohormone responsive category further included salicylic acid (SA) responsive element (TCA-element), gibberellin (GA) responsive elements (GARE-motif, P-box and TATC-box), and auxin responsiveness elements (AuxRR-core and TGA-element) (**Figure 6B**). Among them, the least number of *cis*-element from the phytohormone category was AuxRR-core, which was found in only three *AvLACS* genes. Moreover, the stress response category included *cis*-elements related to biotic and abiotic stress, such as anaerobic induction, wound responsiveness, drought responsiveness, low-temperature responsiveness and defense and stress responsiveness (**Figure 6A**). Notably, almost all the *AvLACS* genes contained anaerobic responsive *cis*-elements, such as ARE and GC-motif, which could respond to hypoxia stress. The expression of *AtLACS2* and LACS enzyme activity are affected by hypoxia, which restricts energy production via aerobic respiration (Zhao et al., 2021; Wundersitz et al., 2025). In contrast, there is only one gene involved in the response to defense and stress, namely, *AvLACS9.2b*.

### 3.7 Expression Analysis of *AvLACS* genes in *A. valvata*

**Figure 7:**
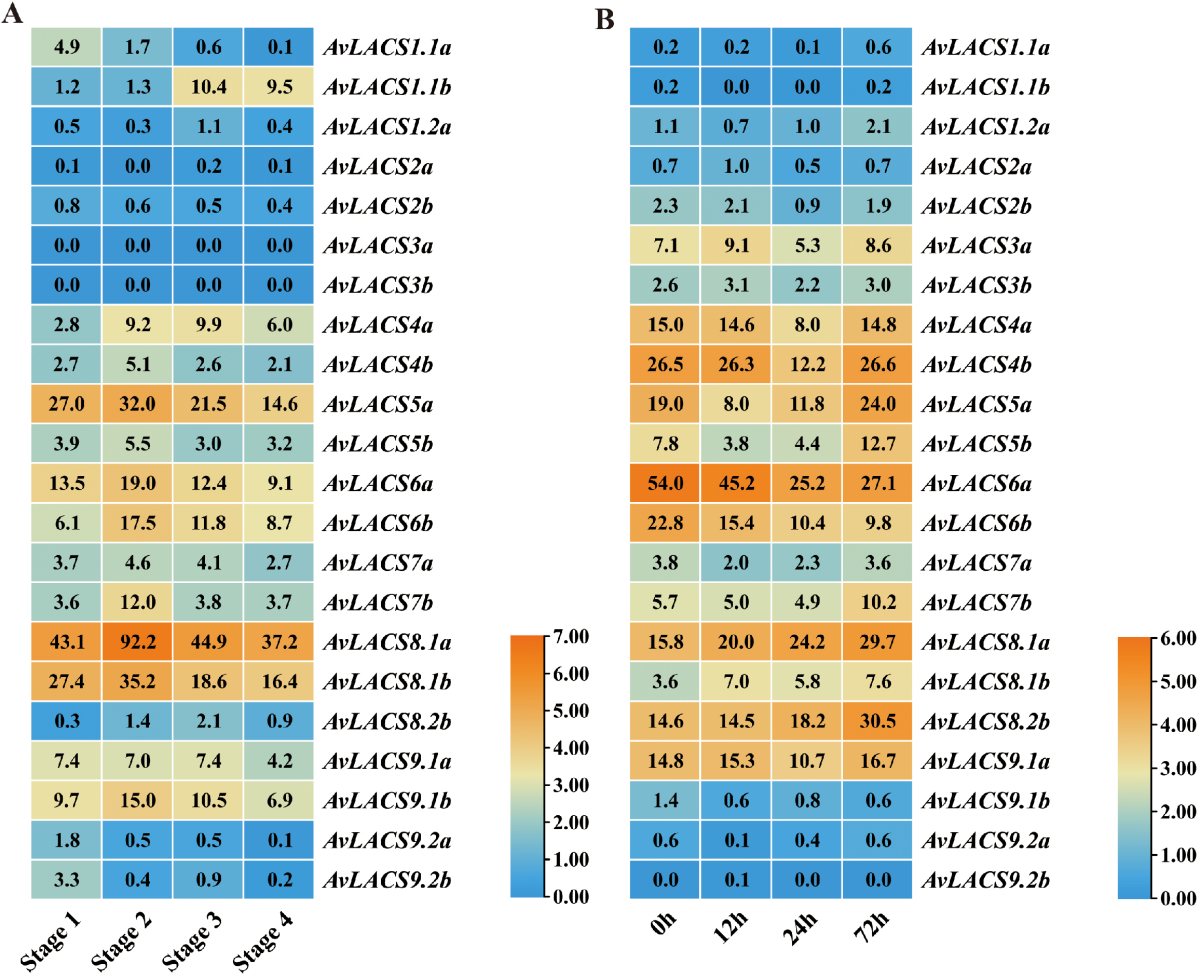
Expression profiles of *AvLACSs* in different fruit stages and under salt stress: **(A)** expression profiles of *AvLACSs* in fruit flesh at stage 1 (mature green fruit stage), stage 2 (breaker fruit stage), stage 3 (color change fruit stage), and stage 4 (ripe fruit stage); **(B)** expression profiles of *AvLACSs* in the roots under salt stress at 0 h, 12 h, 24 h, and 72 h.

To understand the expression patterns of the *AvLACS* genes in *A. valvata*, the RNA-seq data (PRJNA984935) of kiwifruit were extracted and the FPKM (fragments per million mapped readings per thousand base transcripts) were used to evaluate their expression levels in the fruit flesh at different developmental stages. As shown in **Figure 7A**, *AvLACS5a*, *AvLACS6a/b*, *AvLACS8.1a/b* and *AvLACS9.1a/b* were highly expressed in the fruit flesh across four developmental stages – the stage 1 (mature green fruit stage), the stage 2 (breaker fruit stage), the stage 3 (color change fruit stage), and the stage 4 (ripe fruit stage). In contrast, genes like *AvLACS2a/b*, *AvLACS3a/b* and *AvLACS9.2a/b* showed relatively lower expression levels. Additionally, the expression patterns of *AvLACS* family members exhibited differences at different stages of fruit development (**Figure 7A**). The expression levels of *AvLACS1.1a* and *AvLACS9.2a/b* decreased during fruit development, while nearly half of the *AvLACS* family members showed an increase in expression during the breaker fruit stage (stage 2), such as *AvLACS4a/b*, *AvLACS5a/b*, *AvLACS6a/b*, and *AvLACS8.1a/b*, indicating *AvLACSs* may play a key role at this stage.

We further analyzed the expression patterns of *AvLACS* genes in the roots of *A. valvata* under salt stress by utilizing another RNA-seq dataset (PRJNA726156) of kiwifruit. As shown in **Figure 7B**, the *AvLACS4b* and *AvLACS6a/b* were highly expressed in the root under normal conditions. However, the expression levels of *AvLACS6a/b* gradually decreased after the salt stress treatment. In contrast, several genes like *AvLACS7b*, *AvLACS8.1a/b* and *AvLACS8.2b* showed an increase expression after salt treatment for 72 h, suggesting that these genes were involved in regulating response to salt stress, particularly long-time salt stress.

### 3.8 RT-qPCR analysis of *AvLACSs* under waterlogging stress at different times

**Figure 8:**
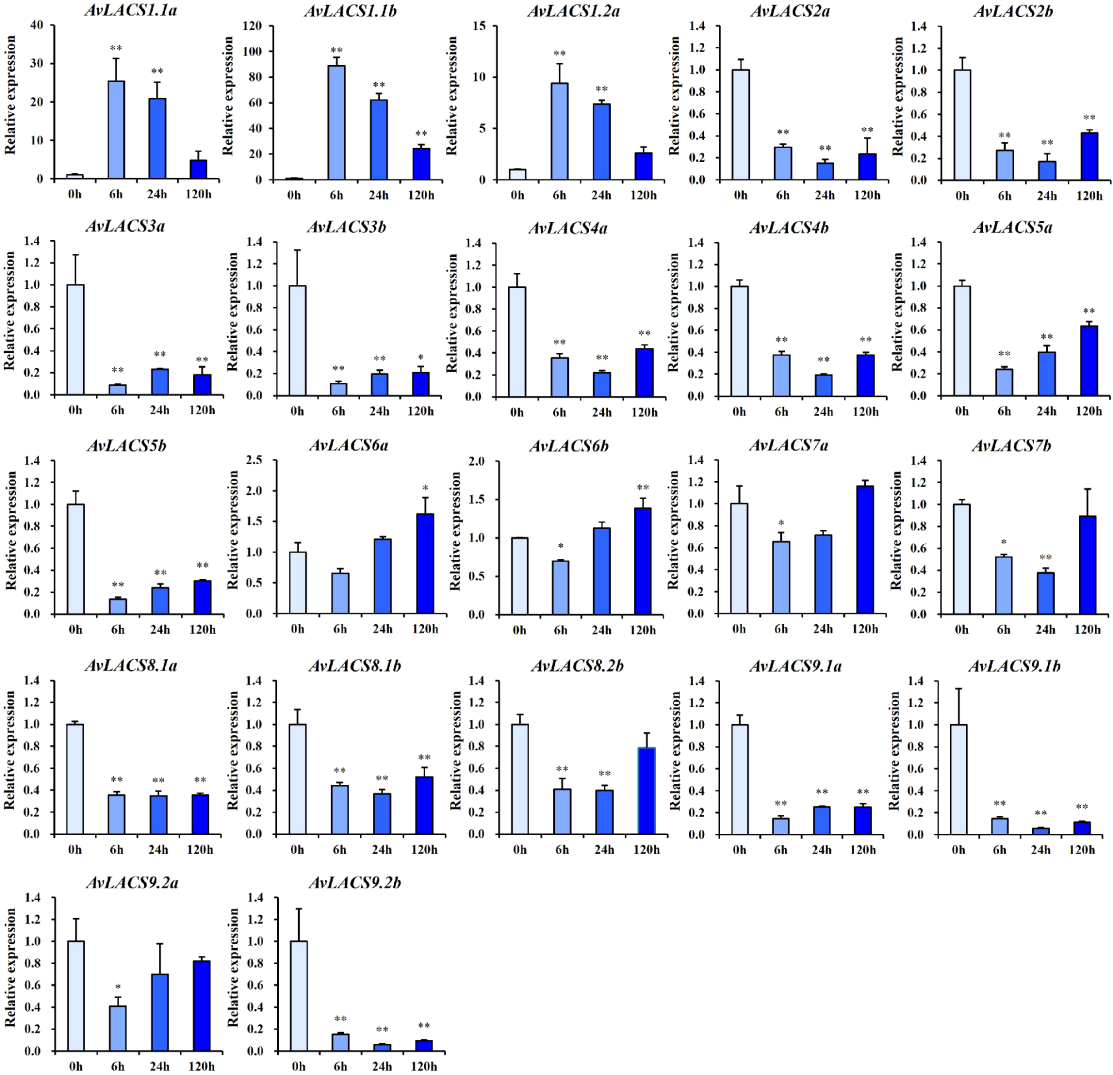
The relative expression profiles of *AvLACSs* in the roots under waterlogging stress at 0 h, 6 h, 24 h, and 120 h. Data are presented as means ± SE (*n* = 3), with statistical significance denoted by *(*p* < 0.05) and **(*p* < 0.01).

To further assess the role of the *AvLACSs* in the response to waterlogging stress, the expression of all of the *AvLACS* genes was measured by real-time quantitative polymerase chain reaction (RT-qPCR). As shown in **Figure 8**, the relative expression levels of nearly all *AvLACS* genes were significantly altered after the waterlogging treatment. Among them, most *AvLACS* genes were downregulated after the waterlogging treatment, such as *AvLACS2a/b*, *AvLACS3a/b*, *AvLACS4a/b*, and *AvLACS5a/b*. The reduction of LACS activity resulted the shift of the composition of LCFAs which triggers the activation of anaerobic metabolism genes (Wundersitz et al., 2025). In contrast, the expression of *AvLACS1.1a/b*, *AvLACS1.2a* and *AvLACS6a/b* increased under the waterlogging stress. Interestingly, the expression pattern of these five genes was different at different treatment time. Of these, *AvLACS1.1a/b* and *AvLACS1.2a* were rapidly induced by the submergence treatment at 6 h and displayed a trend of decline following the waterlogging stress. However, the expression of *AvLACS6a/b* increased significantly until the waterlogging treatment at 120 h. The results suggested that *AvLACS1.1a/b* and *AvLACS1.2a* were crucial for the short-term response, whereas *AvLACS6a/b* might be involved in regulating the long-term response to waterlogging stress.

### 3.9 Expression of cuticular cutin, wax, and TAG biosynthesis genes under waterlogging stress

**Figure 9:**
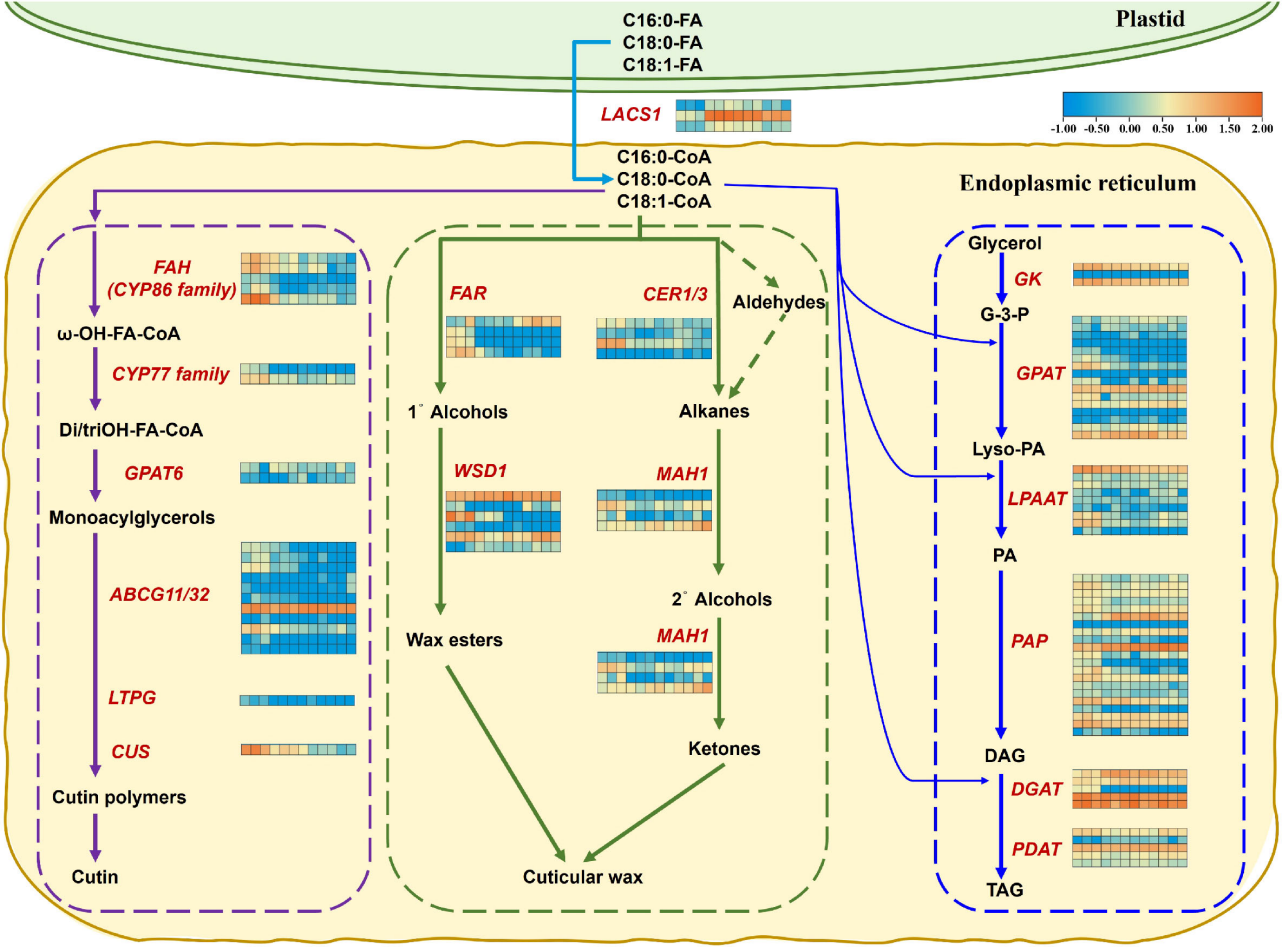
Expression analysis of genes responsible for cuticular cutin, wax, and TAG biosynthesis in the roots of *A. valvata* under waterlogging stress at 0 h, 6 h, 24 h, and 120 h. Purple dotted box indicates genes involved in cuticular cutin biosynthesis, green dotted box indicates genes involved in cuticular wax biosynthesis, and blue dotted box indicates genes involved in TAG biosynthesis. Transcript IDs for the heatmap are listed in the corresponding Supplementary materials files. Heatmaps were generated using the transcriptome data using the TBtools, and orange and blue colors in the heatmaps indicate higher and lower abundances, respectively. The twelve squares in each horizontal row represent the twelve samples (0 h-1, 0 h-2, 0 h-3, 6 h-1, 6 h-2, 6 h-3, 24 h-1, 24 h-2, 24 h-3, 120 h-1, 120 h-2 and 120 h-3). Abbreviations used in the figure: LACS1, long chain acyl-CoA synthetase 1; FAH, fatty acyl ω-hydroxylase; CYP86 family, cytochrome P450-CYP86 family; CYP77 family,cytochrome P450-CYP77 family; GPAT6, glycerol-3-phosphate acyl-transferase 6; ABCG11/32, ABC transporter G family member 11 and 32; LTPG, GPI-anchored lipid transfer protein; CUS, cutin synthase; FAR, fatty acyl-CoA reductase; WSD1, bifunctional wax ester synthase/acyl-CoA: diacylglycerol acyltransferase 1; CER1/3, very-long-chain aldehyde decarbonylase 1 and 3; MAH1, midchain alkane hydroxylase 1; GK, glycerol kinase; LPAAT, lysophosphatidic acid acyltransferase; PAP, phosphatidate phosphatase; DGAT, diacylglycerol acyl-transferase; PDAT, phospholipid: diacylglycerol acyltransferase; FA, fatty acid; CoA, coenzyme A; G-3-P, glycerol 3-phosphate; Lyso-PA, lysophosphatidic acid; PA, phosphatidic acid; DAG, diacylglycerol; TAG, triacylglycerol.

To understand the metabolic pathway that AvLACS1 (AvLACS1.1a/b and AvLACS1.2a) will subsequently participate in, the expression patterns of related genes involved in these pathways under waterlogging stress were analyzed based on the transcriptomic data (**Figure 9**). In Arabidopsis, AtLACS1 located in ER is prominently involved in several fatty acid-derived metabolic pathways, including the synthesis of wax, cutin, and TAG. Given that AvLACS1.1a/b and AvLACS1.2a are predicted to be located in the ER, and they exhibit a close evolutionary relationship with AtLACS1, it is hypothesized that AvLACS1 (AvLACS1.1a/b and AvLACS1.2a) may be involved in the synthesis of wax, cutin, and TAG. As shown in **Figure 9**, LCFAs were transferred to the ER and converted to acyl-CoA by AvLACS1. Under waterlogging stress, the expression levels of *AvLACS1.1a/b* and *AvLACS1.2a* rapidly increased at 6 h of treatment, and then gradually decreased, which is consistent with our qRT-PCR results. Subsequently, the synthesized acyl-CoA can be directed into three metabolic pathways: cutin biosynthesis, cuticular wax biosynthesis and TAG biosynthesis. In the cutin biosynthesis pathway, the expression of most genes was downregulated after waterlogging treatment (**Figure 9**; **Supplementary Table 6**). In the cuticular wax biosynthesis pathway, four *FAR* genes, six *WSD1* genes, four *CER1/3* genes and four *MAH1* genes were identified (**Figure 9**; **Supplementary Table 6**). Among them, three *FAR* genes were downregulated after waterlogging treatment, while one *FAR* gene was upregulated until 120 h of treatment. In the *WSD1* gene family, the expression of a *WSD1* gene gradually increased after waterlogging treatment, another *WSD1* gene showed an initial decrease followed by an increase in expression, and the expression of one *WSD1* gene was downregulated after treatment. In another pathway involved in cuticular wax precursor synthesis, the expression of two *CER1/3* genes was downregulated after treatment, while one *MAH1* gene exhibited an initial decrease followed by an increase in expression after treatment, and one gene showed a gradual increase in expression in response to waterlogging stress. In the TAG biosynthesis pathway, one *GPAT* gene, one *PAP* gene and one *DGAT* gene rapidly increased at 6 h of treatment, and then gradually decreased, which is consistent with the expression patterns of *AvLACS1.1a/b* and *AvLACS1.2a* (**Figure 9**; **Supplementary Table 7**). Based on the transcription levels of related biosynthetic genes in these three metabolic pathways, it is inferred that acyl-CoA synthesized by AvLACS1 is primarily utilized in the synthesis of cuticular wax and TAG.

### 3.10 Expression of TAG degradation pathway genes under waterlogging stress

**Figure 10:**
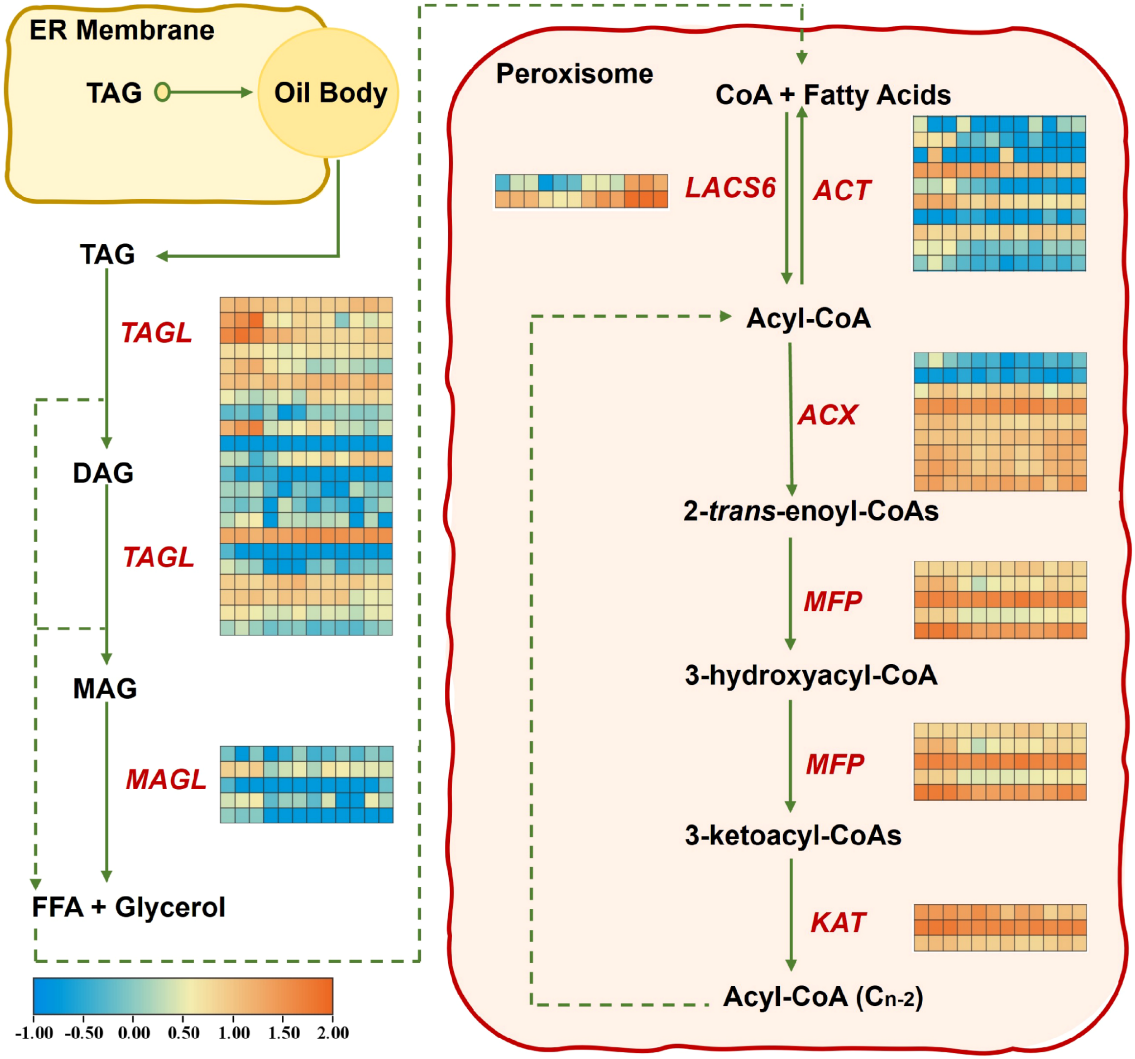
Expression analysis of genes related to TAG degradation in the roots of *A. valvata* under waterlogging stress at 0 h, 6 h, 24 h, and 120 h. Transcript IDs for the heatmap are listed in the corresponding Supplementary Materials file. Heatmaps were generated using the transcriptome data using TBtools, and orange and blue colors in the heatmaps indicate higher and lower abundances, respectively. The twelve squares in each horizontal row represent the twelve samples (0 h-1, 0 h-2, 0 h-3, 6 h-1, 6 h-2, 6 h-3, 24 h-1, 24 h-2, 24 h-3, 120 h-1, 120 h-2 and 120 h-3). Abbreviations used in the figure: TAGL, triacylglycerol lipase; MAGL, monoacylglycerol lipase; LACS6, long chain acyl-CoA synthetase 6; ACT, acyl-CoA thioesterase; ACX, acyl-CoA oxidase; MFP, multifunctional protein; KAT, 3-ketoacyl-CoA thiolase; ER, endoplasmic reticulum; TAG, triacylglycerol; DAG, diacylglycerol; MAG, monoacylglycerol; FFA, free fatty acid; CoA, coenzyme A.

Considering that AvLACS6a/b are located in the peroxisome and that phylogenetic analysis reveals a close evolutionary relationship to AtLACS6, it is hypothesized that AvLACS6a/b may mediate TAG degradation through fatty acid β-oxidation like AtLACS6. The fatty acid β-oxidation pathway is summarized in **Figure 10**, and the expression patterns of genes under waterlogging stress associated to this pathway were presented as heatmap. During β-oxidation, twenty-two *AvTAGL* genes were identified to be differentially expressed under waterlogging stress at different times, and two of them were relatively upregulated with the extension of treatment time (**Figure 10**; **Supplementary Table 8**). Five *AvMAGL* genes were identified and one of them was downregulated rapidly after the waterlogging treatment at 6 h and then gradually upregulated at 120 h. Subsequently, the FFAs are then transported to peroxisomes. In peroxisomes, acyl-CoA is synthesized from the FFAs by AvLACS6. Similar to our qRT-PCR results, *AvLACS6a* and *AvLACS6b* were both upregulated under waterlogging stress at 120 h. In contrast, ten *AvACT* genes were identified and two of them were downregulated under waterlogging stress. The acyl-CoAs are first converted to 2-*trans*-enoyl-CoAs by ACX, which are subsequently processed through 3-hydroxyacyl-CoAs by MFP to form 3-ketoacyl-CoAs (Yoshitake et al., 2019). A total of eight *AvACX* genes were identified, three of which were downregulated at the initial stage of waterlogging treatment and subsequently upregulated at 120 h. Two *AvMFP* genes exhibited similar trends in expression with the aforementioned three *AvACX* genes. At the last step, the 3-ketoacyl-CoAs are then hydrolyzed to produce acyl-CoAs by KAT, and the hydrolyzed acyl-CoA is used as substrates for acyl-CoA oxidases. Three *AvKAT* genes were identified and all of them downregulated after waterlogging treatment. Based on the transcription levels of related biosynthetic genes in the TAG degradation pathway, at the long-term waterlogging stress, the esterification of fatty acids to acyl-CoAs by AvLACS6a/b results in their activation for oxidative attack at the β-carbon position until the complete degradation of long-chain acyl-CoAs to C_2_ acetyl units.

### 3.11 Lipids profiling in the roots of *A. valvata* under waterlogging stress

**Figure 11:**
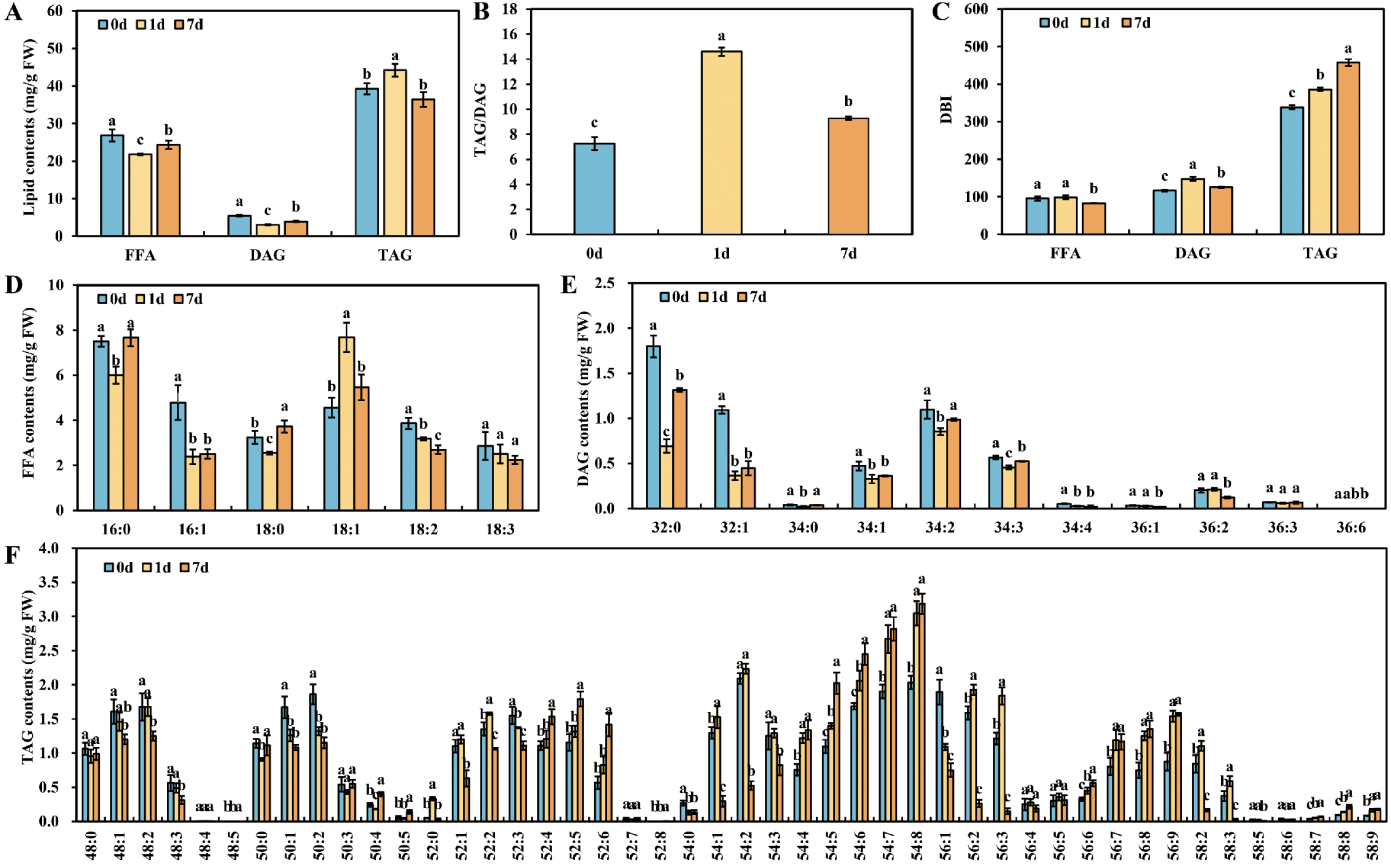
The composition and content of lipids in roots of *A. valvata* under waterlogging stress: **(A)** the lipid content of FFA, DAG and TAG in the roots under waterlogging stress at 0 d, 1d and 7 d; **(B)** the TAG/DAG ratio in the roots under waterlogging stress at 0 d, 1d and 7 d; **(C)** the DBI of FFA, DAG and TAG in the roots under waterlogging stress at 0 d, 1d and 7 d; **(D–F)** fatty acid composition of FFA **(D)**, DAG **(E)** and TAG **(F)** in the roots under waterlogging stress at 0 d, 1d and 7 d. FFA, free fatty acids; DAG, diacylglycerol; TAG, triacylglycerol; DBI, double bond index. Values are presented as means ± standard deviation, different letters indicate significant differences at the *p* < 0.05 level.

To further validate our hypothesis regarding the metabolic pathways involving AvLACS1 and AvLACS6, the FFA, DAG and TAG from the roots of *A. valvata* at 0, 1and 7 days of waterlogging treatment were measured and identified by LC-MS/MS. As shown in **Figure 11A**, the content of FFA and DAG decreased significantly after submergence treatment, and although FFA and DAG showed some recovery after seven days of waterlogging, their levels remained significantly lower than those of the control. By contrast, the content of TAG accumulated significantly at 1 day of submergence treatment, while it returned to the normal level at 7 days. Meanwhile, the TAG/DAG ratio increased sharply at 1 day of submergence treatment and then decreased slightly at 7 days, but their levels were significantly higher than those of the control group (**Figure 11B**). The changes in the contents of these three lipids and the TAG/DAG ratio are consistent with our previous hypothesis that short-term waterlogging stress mediates TAG synthesis through AvLACS1, while long-term waterlogging stress mediates TAG degradation through AvLACS6. In addition, the double bond index (DBI), which indicates the degree of unsaturation, of FFA, DAG and TAG was calculated (**Figure 11C**), and the DBI of TAG was relatively large compared to that of FFA and DAG. Under waterlogging stress, the DBI of FFA decreased with the prolongation of submergence treatment, while the DBI of DAG and TAG increased significantly during submergence treatment. Significant changes in the lipid composition were observed in the roots of *A. valvata* with prolonged waterlogging stress. Waterlogging treatment induced a significant decrease in the content of various fatty acids in FFA and DAG, however, the content of the saturated FFA (16:0-FFA and 18:0-FFA) and DAG (32:0-DAG, 34:2-DAG and 34:3-DAG) gradually increased at 7 days of waterlogging stress (**Figures 11D, E**). In contrast, the content of the polyunsaturated TAG increased under waterlogging stress in parallel with the decreasing of less unsaturated TAG (**Figure 11F**), indicating that TAG serves as a pool for polyunsaturated fatty acids (Lu et al., 2020), and the incorporation of polyunsaturated fatty acids into TAG during waterlogging stress could be an important mechanism of plant adaptation to waterlogging stress.

## 4 Discussion

Long-chain acyl-CoA synthetase (LACS) plays a pivotal role in fatty acid metabolism and catabolism by converting free fatty acids to acyl-CoAs (Zhao et al., 2021). The generated acyl-CoAs serve as substrates for the synthesis of various lipids, including membrane lipids, cuticular lipids and TAG, which are not only essential for normal plant growth, but also are crucial for plant adaption to environmental stresses, such as waterlogging stress (Kęska et al., 2021; Zhou et al., 2020; Xie et al., 2020). As a waterlogging-tolerant kiwifruit, an increasing number of recent studies on *A. valvata* have focused on the adaptive strategies employed by *A. valvata* under waterlogging stress. Previous studies reported that four DEGs encoding LACS were significantly upregulated under waterlogging stress in *A. valvata* (Li et al., 2022). However, systematic identification and characterization of the *LACS* gene family in *A. valvata* is lacking to date. In this study, we identified 22 *AvLACS* members from the genome of *A. valvata*, which were located on 21 chromosomes. The number of *AvLACS* genes identified in the *A. valvata* genome was higher than the numbers found in *A. thaliana* (11 *AtLACSs*) (Shockey et al., 2002), *Z. mays* (11 *ZmLACSs*) (Wang et al., 2023), *M. domestica* (11 *MdLACSs*) (Zhang et al., 2018), *G. max* (17 *GmLACSs*) (Wang et al., 2022) and *C. illinoinensis* (11 *CiLACSs*) (Ma et al., 2023), but less than those in *T. aestivum* (30 *TaLACSs*) (Liu et al., 2022) and *B. napus* (34 *BnaLACSs*) (Xiao et al., 2019), which may be due to the differences in the sizes of their genomes. The *AvLACSs* encoded proteins ranging from 478 to 734 amino acids, and these proteins were located in the endoplasmic reticulum, peroxisome, and chloroplast. The subcellular localization of the AvLACSs was similar to the LACSs from other plants (Shockey et al., 2002; Liu et al., 2022; Zhou et al., 2024).

The multiple sequence alignment of AvLACS and AtLACS confirmed that AvLACS protein sequences contained two conserved motif domains (AMP-binding domain signature and ACS signature motif), which is consistent with CiLACS (Ma et al., 2023). Moreover, the AvLACS proteins were classified into five clusters based on phylogenetic tree analysis, and similar subcellular localization, exon-intron numbers, motif compositions, and protein structure were found within each cluster. The gene structure of *AvLACS* members in cluster II and III contained 19 exons and 18 introns, which is consistent with the number of exons found in the same clusters in *C. illinoinensis* (Ma et al., 2023). The conserved exon-intron structure in cluster II and III indicates that the *LACS* genes may have originated from a common ancestor, and have been significantly influenced by recurrent gene duplication events throughout their evolutionary history (Lynch et al., 2001). The greatest number of exons were found in *AvLACS6a/b* and *AvLACS7a/b* in cluster IV, which contained 23 exons. It is consistent with those of *AtLACS6* and *AtLACS7* in Arabidopsis (Shockey et al., 2002), *MdLACS6.1* and *MdLACS6.2* in *M. domestica* (Zhang et al., 2018), *BnaLACS6-1/2/3/4* and *BnaLACS7-1/2* in *B. napus* (Xiao et al., 2019), *CiLACS6*, *CiLACS6–1* and *CiLACS7* in *C. illinoinensis* (Ma et al., 2023). These variant exon-intron structures of *AvLACS* genes in different clusters suggested that *AvLACS* gene family may possess diverse functions, as it has been reported that the divergences in exon-intron structures can lead to changes of gene function (Xu et al., 2012).

The functional diversification of *AvLACS* genes is also reflected in the diversity of *cis*-acting elements in their promoter regions. In our study, *cis*-acting elements in the promoter regions of 22 *AvLACS* genes were predicted and classified into three subfamilies, including plant growth and development, phytohormone responsive, and stress-responsive subfamilies. Among them, the phytohormone-responsive *cis*-acting elements that are widely distributed include MeJA and ABA responsive elements, as well as elements responsive to SA, GA, and auxin. Some of the predictions were confirmed by the expression analysis, for instance, the expression level of *PoLACS4* were significantly downregulated in mature leaves treated with 100 μmol/L ABA (Zhang et al., 2022), while the expression of *MdLACS* genes was upregulated with ABA treatment (Zhang et al., 2018). In addition, the stress response category contained *cis*-elements related to biotic stress such as defense and stress responsiveness, and to abiotic stress, such as anaerobic induction, wound responsiveness, drought responsiveness, and low-temperature responsiveness. Under biotic stress, loss of function of AtLACS2 could increase resistance to *Botrytis cinerea* (Bessire et al., 2007) and susceptibility to avirulent *Pseudomonas syringae* (Tang et al., 2007). Under abiotic stress, the expression of *GmLACS9/15/17* was significantly upregulated under alkali, low temperature and drought stress (Wang et al., 2022). Similarly, several *MdLACS* genes were significantly upregulated under drought stress (Zhang et al., 2018), as the LACSs are crucial in the synthesis of cuticular lipids, and the formed cuticular can serve as surface barriers to prevent further water loss under drought stress (Zhao et al., 2021; Weng et al., 2010). The accumulation of cuticular wax enhanced the resistance of *MdLACS2* or *MdLACS4* transgenic plants to drought and salt stress (Zhang et al., 2020a, Zhang et al., 2020b). Furthermore, the expression of *LACS6* in cucumber and four *LACSs* in kiwifruit (*A. valvata*) were significantly upregulated under waterlogging stress (Li et al., 2022; Kęska et al., 2021). AvLACSs might play an important role in *A. valvata* under waterlogging stress, as the anaerobic responsiveness *cis*-element was widely distributed in the promoter regions of *AvLACSs*.

To further elucidate the function of the *AvLACS* genes, we analyzed the expression patterns of *AvLACSs* during different stages of fruit development and under salt stress using transcriptome data available from NCBI. Based on the transcriptome data, nearly half of the *AvLACS* family members showed an increase expression at the breaker fruit stage, such as *AvLACS4a/b*, *AvLACS5a/b*, *AvLACS6a/b*, and *AvLACS8.1a/b*. Fleshy fruits are covered by the cuticle, which has an important protective role during fruit development and ripening (Trivedi et al., 2019). To prevent fruit softening and enhance the resistance to pathogens or water loss, the cuticular wax load increases during fruit development leading to a thick cuticle at maturity (Trivedi et al., 2019). The upregulated expression of these *AvLACS* genes at the breaker fruit stage might play a role in the synthesis of fruit cuticular wax to protect the fruit against the pathogens and water loss. Moreover, the expression levels of *LACS* genes under salt stress were different at various treatment times. The expression of *AvLACS6a/b* gradually decreased after salt stress treatment. Similarly, the expression levels of most of the *MdLACS* genes decreased under salt treatment in apple (Zhang et al., 2018). In pecan, the expression levels of *CiLACS1*, *CiLACS1-1*, and *CiLACS4–1* were down-regulated under salt stress (Ma et al., 2023). However, *AvLACS7b*, *AvLACS8.1a/b* and *AvLACS8.2b* showed an increased expression after salt treatment for 72 h. The expression levels of *CiLACS6*, *CiLACS7*, *CiLACS9*, and *Ci-LACS9–1* were upregulated after salt treatment at 8 and 16 days (Ma et al., 2023). These results indicate that LACSs play a crucial role in regulating response to salt stress, particular long-time salt stress.

In addition to their roles in drought and salt stress, increasing evidence suggests that LACSs are involved in plant response to waterlogging stress. In Arabidopsis, AtLACS2 is a key enzyme for wax and cutin biosynthesis, and the transgenic lines overexpressing *LACS2* displayed enhanced resistance to submergence by modulating the cuticle integrity and permeability (Xie et al., 2020), as well as modulating the translocation of the ERF-VII transcription factor from the membrane to the nucleus (Zhou et al., 2020). In our study, the expression of all *AvLACS* genes was measured by RT-qPCR, and the results showed that the expression of *AvLACS1.1a/b*, *AvLACS1.2a* and *AvLACS6a/b* increased significantly after waterlogging treatment. Similarly, the expression level of *LACS6* in cucumber exhibited a significant increase in response to waterlogging treatment (Kęska et al., 2021). Moreover, the expression of *AvLACS1.1a/b* and *AvLACS1.2a* was rapidly upregulated at 6 h, suggesting their involvement in the immediate response to short-term waterlogging stress. In contrast, *AvLACS6a/b* likely regulates the long-term adaptation to waterlogging, as its expression progressively increased up to 120 h of submergence treatment.

To elucidate the distinct mechanisms of different *AvLACS* genes in short-term or long-term waterlogging responses, transcriptomic analysis was performed to examine the expression profiles of associated genes within relevant metabolic pathways. During the short-term waterlogging response, the data suggest that acyl-CoA synthesized by AvLACS1 is primarily utilized in the synthesis of wax and TAG. Immediately after waterlogging, the rapid synthesis of wax might help plants regulate cuticle permeability, thereby regulating water transmission, improving gas exchange, and enhancing root resistance to pathogens caused by hypoxia (Xie et al., 2021). Homologs of *AvLACS1*, such as *SlLACS1* in tomato has been demonstrated to play a crucial role in wax biosynthesis (Wu et al., 2024). Simultaneously, TAG serves as a safe transient reservoir of acyl groups, and its rapid accumulation plays a pivotal role in buffering lipid homeostasis and protecting cells against lipotoxic death by FA overload, which often results from extensive membrane remodeling during the stress response (Wang et al., 2019; He and Ding, 2020). Our lipid analysis of *A. valvata* roots under waterlogging stress confirmed the hypothesis that TAG plays a critical role in stress adaptation, demonstrating that FFA and DAG levels significantly decreased, likely due to their rapid conversion into TAG, while both the content and the unsaturated level of TAG significantly increased during short-term waterlogging stress. The increase of TAG content and the degree of polyunsaturation were also observed in tomato roots under hypoxia stress by waterlogging for 48 h (Striesow et al., 2024). Similarly, lipid droplets in the leaves of ramie (*Boehmeria nivea* L.) increased and aggregated in the cytoplasmic in response to submergence stress (Shao et al., 2024). The incorporation of polyunsaturated fatty acids into TAG, leading to increased TAG content and unsaturation, might be a key mechanism of plant adaptation to waterlogging stress.

At the long-term waterlogging response, AvLACS6a/b may mediate TAG degradation through fatty acid β-oxidation in peroxisome, similar to *AtLACS6*. The analysis of lipid composition in roots under long-term waterlogging has provided valuable insights into lipid metabolism during waterlogging stress conditions. The gradual decrease in TAG content is coupled with significant increases in FFA and DAG levels, which strongly supports the hypothesis that TAG undergoes degradation under prolonged waterlogging stress. During the long-term waterlogging stress, the liberation of fatty acids stored in cytoplasmic lipid droplets by TAG degradation can provide fatty acids to facilitate membrane reconstruction (Yu et al., 2021), and then maintain intracellular lipid homeostasis (Wang et al., 2019). Notably, the unsaturation degree of TAG remained unchanged, indicating that TAG continues to serve as a reservoir for polyunsaturated fatty acids even under prolonged waterlogging conditions. Thus, we proposed and have verified that the *AvLACS* gene family in the tetraploid *A. valvata* genome enhances the tolerance of kiwifruit to waterlogging stress through multiple pathways, including the short-term regulation of wax and TAG synthesis, as well as the long-term regulation of TAG degradation during prolonged waterlogging stress. This differential temporal stress-response strategy through regulation of LACS activity affecting TAG metabolism and wax synthesis in *A. valvata* improves survival rate under both short- and long-term waterlogging stress.

## 5 Conclusion

In this study, a total of 22 *AvLACS* genes were identified and systematically analyzed in *A. valvata*. *AvLACSs* were divided into five clusters based on a phylogenetic tree, and similar subcellular localization, exon-intron structures, motif compositions and protein structures were found within each phylogenetic cluster. Collinearity analysis identified 22 duplicated gene pairs in the *A. valvata* genome, which have undergone purifying selection during evolution. Expression profiling revealed that *AvLACS1.1a/b*, *AvLACS1.2a*, and *AvLACS6a/b* were significantly upregulated at specific time points after waterlogging treatment, indicating that the *AvLACS* gene family in the tetraploid *A. valvata*’ genome contributes to enhancing kiwifruit tolerance to waterlogging stress through multiple regulatory pathways. In the initial phase of waterlogging, *AvLACS1.1a/b* and *AvLACS1.2a* are involved in regulating wax and TAG synthesis, which is vital for maintaining cuticle permeability, preserving lipid homeostasis, and enhancing pathogen resistance. Conversely, *AvLACS6a/b* are pivotal during prolonged waterlogging, facilitating TAG degradation through fatty acid β-oxidation to maintain membrane integrity and intracellular lipid stability under extended stress. This coordinated regulation between *AvLACS1*(*AvLACS1.1a/b* and *AvLACS1.2a*) and *AvLACS6a/b* facilitates dynamic lipid metabolic adaptation, enabling *A. valvata* to adapt to both early and sustained phases of waterlogging stress. Integrated lipid profiling and transcriptomic analyses validate that *AvLACS1.1a/b* and *AvLACS1.2a* mediate wax and TAG synthesis, whereas *AvLACS6a/b* are involved in TAG degradation via β-oxidation. These findings, supported by combined lipidomic and transcriptomic data, enhance our understanding of lipid metabolism in *A. valvata* and identify potential molecular targets for breeding waterlogging-tolerant kiwifruit and other crops. Overall, this study elucidates the distinct functional roles of *LACS* family members in kiwifruit, providing a foundation for future studies on the molecular mechanisms of *AvLACS* genes.

## Data Availability

The original contributions presented in the study are included in the article/**Supplementary Material**. Further inquiries can be directed to the corresponding author.
