# Supplementary material for: Identification and functional analysis of AvLACS genes unveils their role in lipid homeostasis and waterlogging tolerance in kiwifruit (Actinidia valvata Dunn)
Source: Front Plant Sci. 2025 Jun 30;16:1580003. doi: 10.3389/fpls.2025.1580003 (PMC12256519; doi:10.3389/fpls.2025.1580003)
Supplement: Supplementary file 1 [file DataSheet1.pdf]

# *Supplementary Material*

## Supplementary Figures

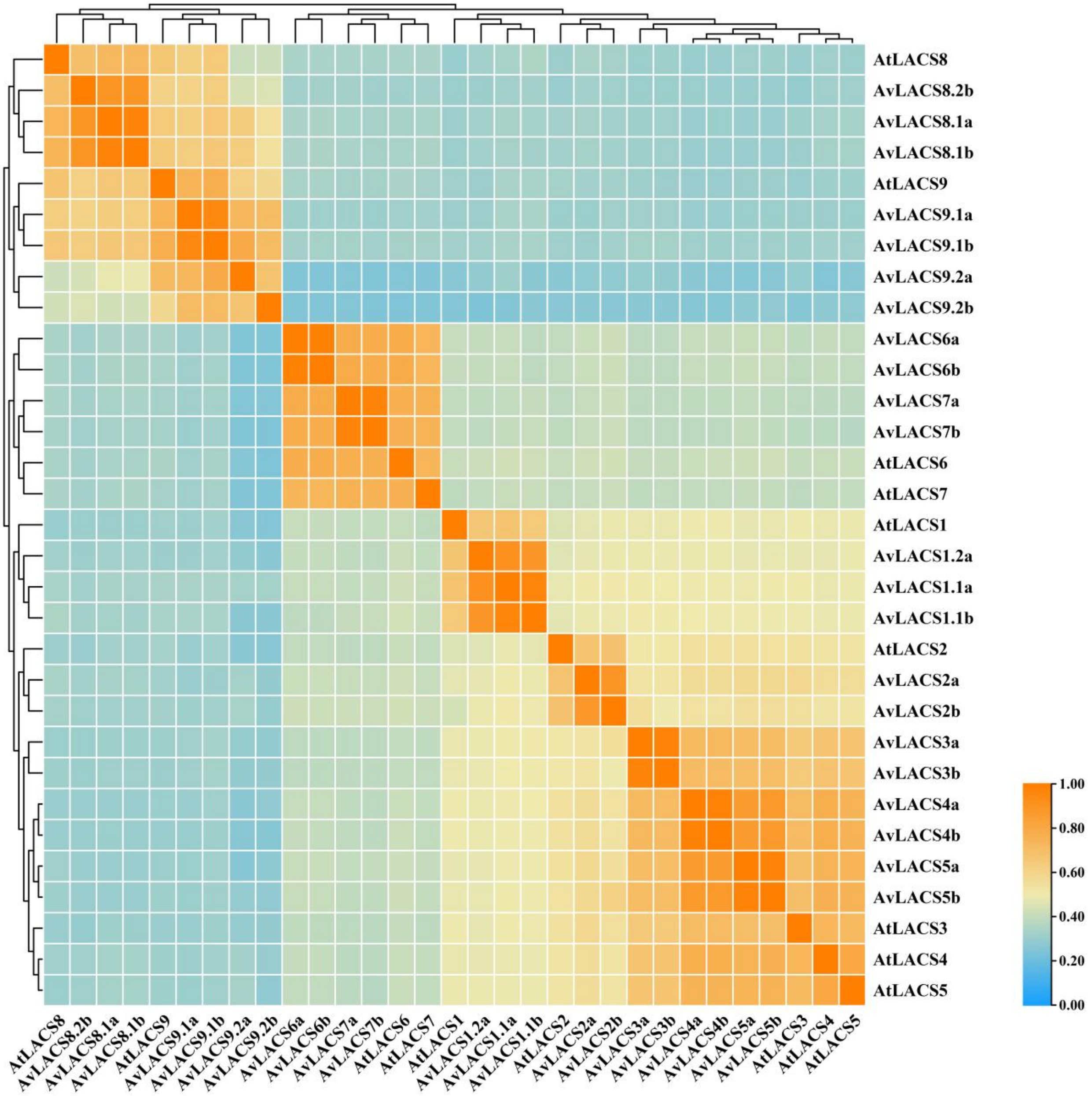


**Supplementary Figure 1**. Amino acid similarity analysis of the AvLACS proteins with AtLACS from *A. thaliana*.

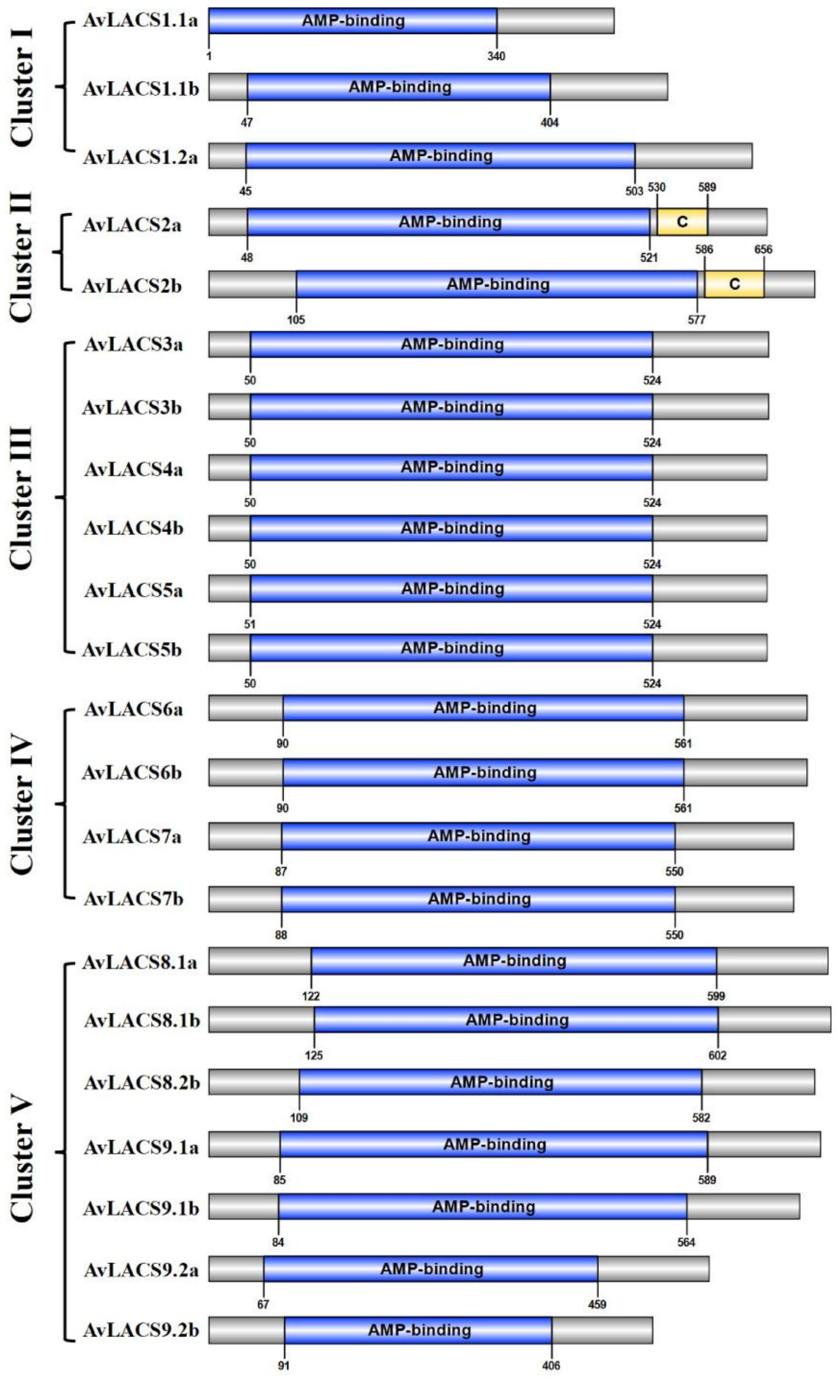


**Supplementary Figure 2**. Analysis of the conserved domains of AvLACS proteins. The numerical markers along the protein sequence indicate the locations of these domains. The AMP-binding C in AvLACS2a/b, which belongs to Cluster II, is marked as ‘C’ (yellow box).

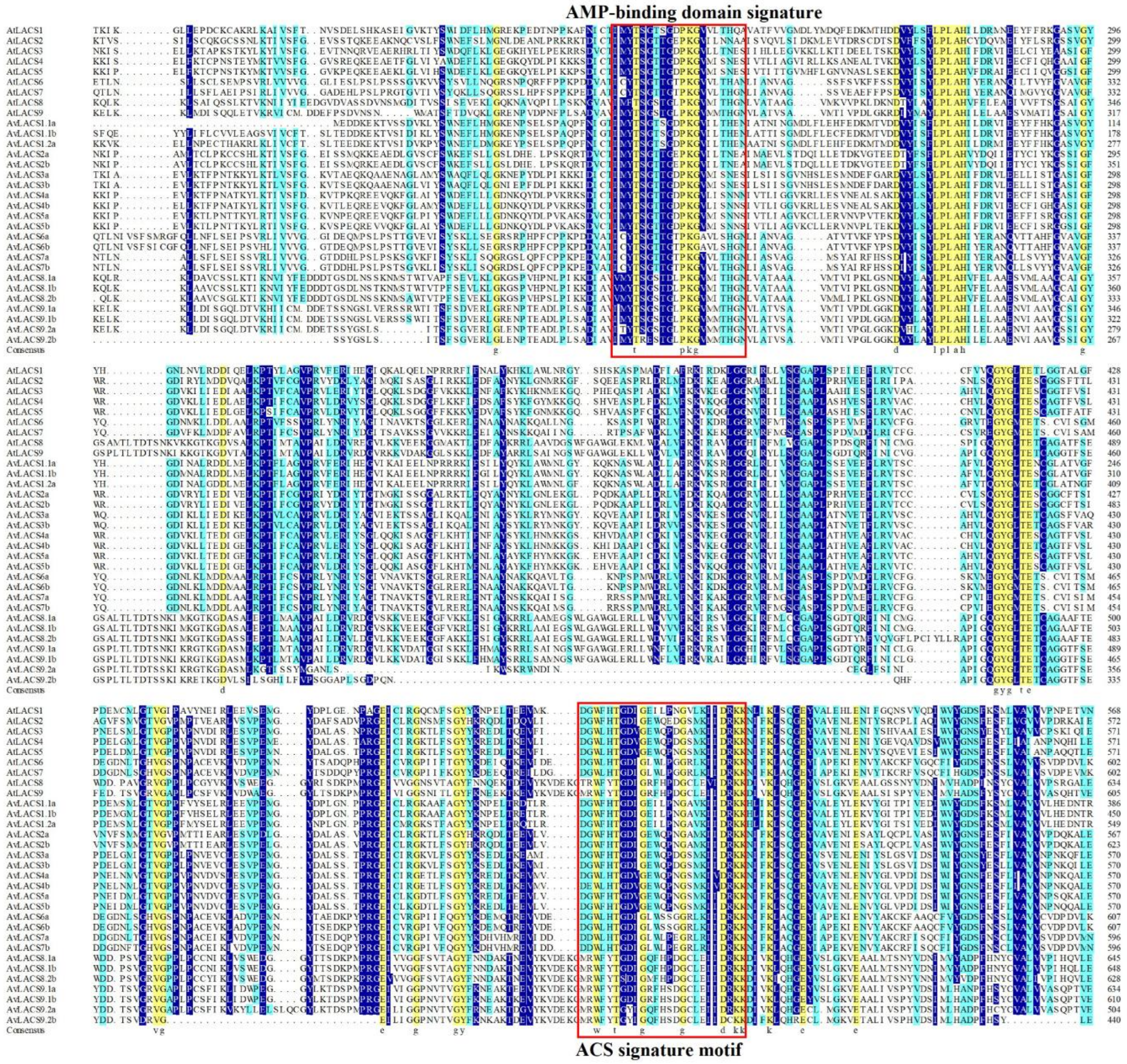


**Supplementary Figure 3**. Multiple sequence alignment of the AvLACS and the AtLACS proteins. Sequence alignment was conducted using DNAMAN 8.0. The distribution of two conserved motifs (the AMP-binding domain signature motif and the ACS signature motif) is marked with a red box.

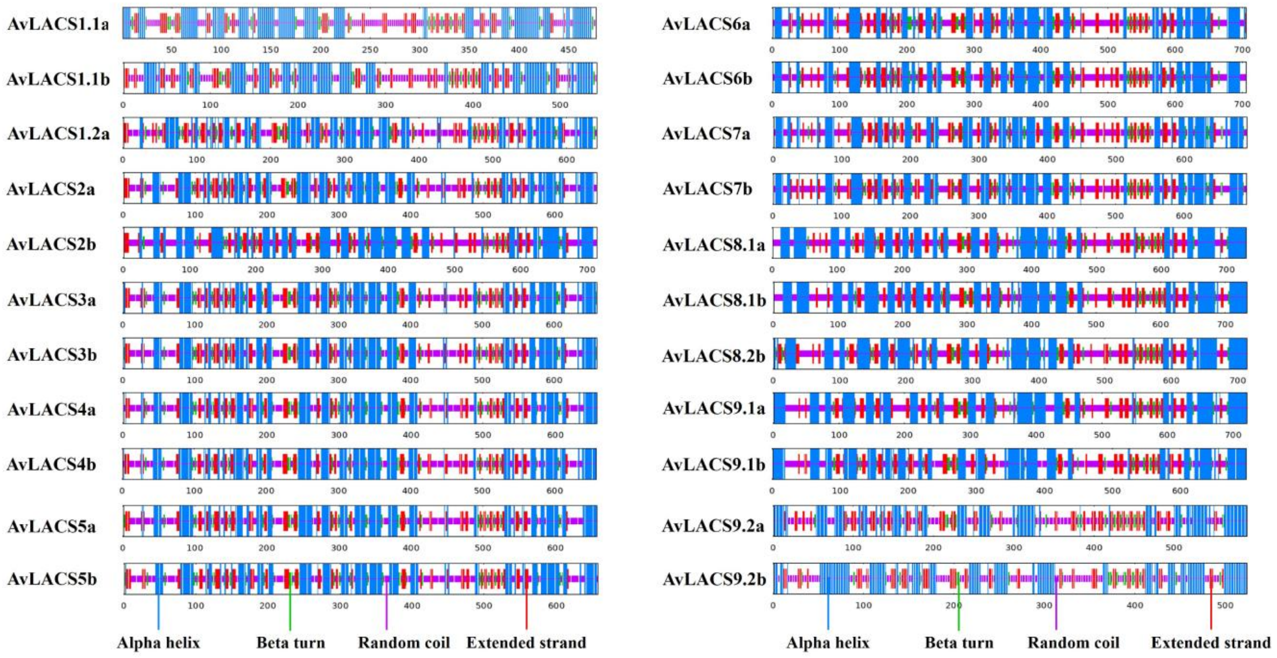


**Supplementary Figure 4**. The secondary structure of the AvLACSs.

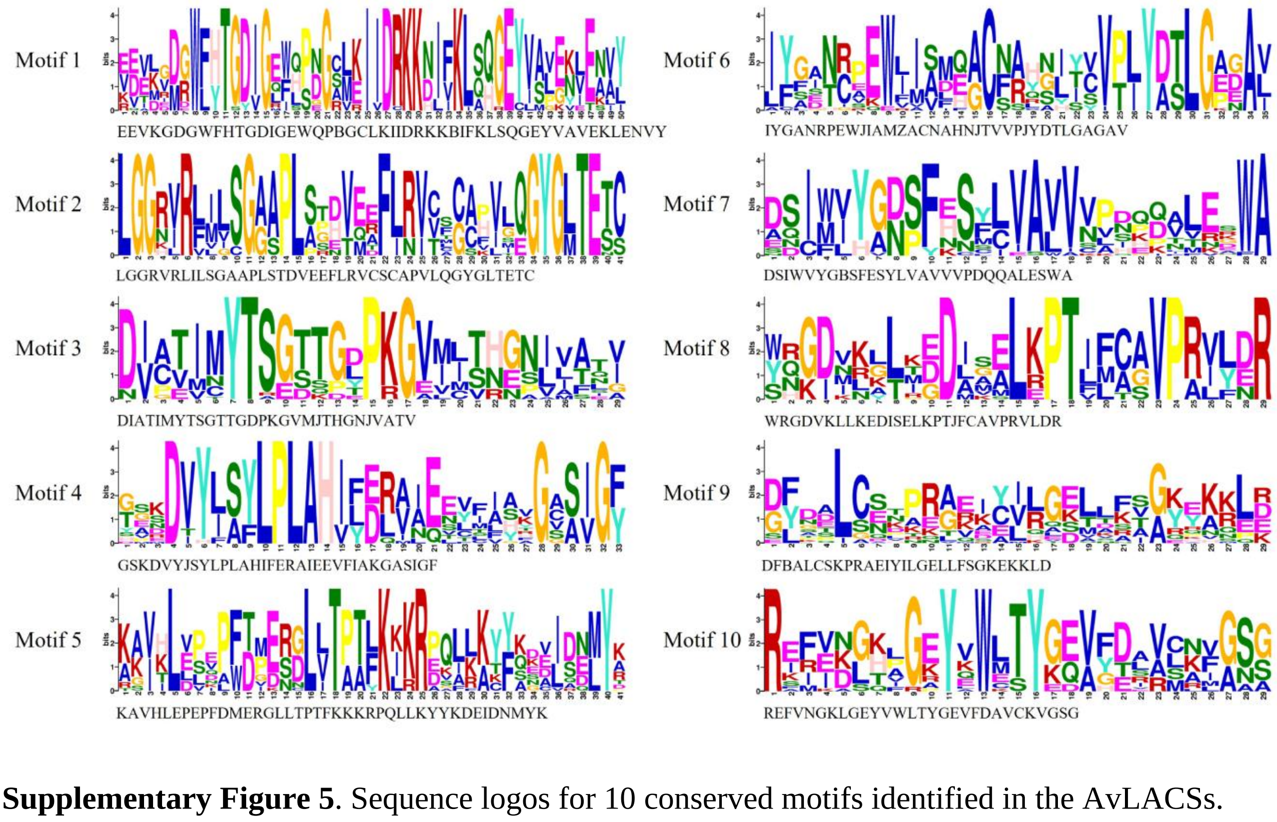


**Supplementary Figure 5**. Sequence logos for 10 conserved motifs identified in the AvLACSs.

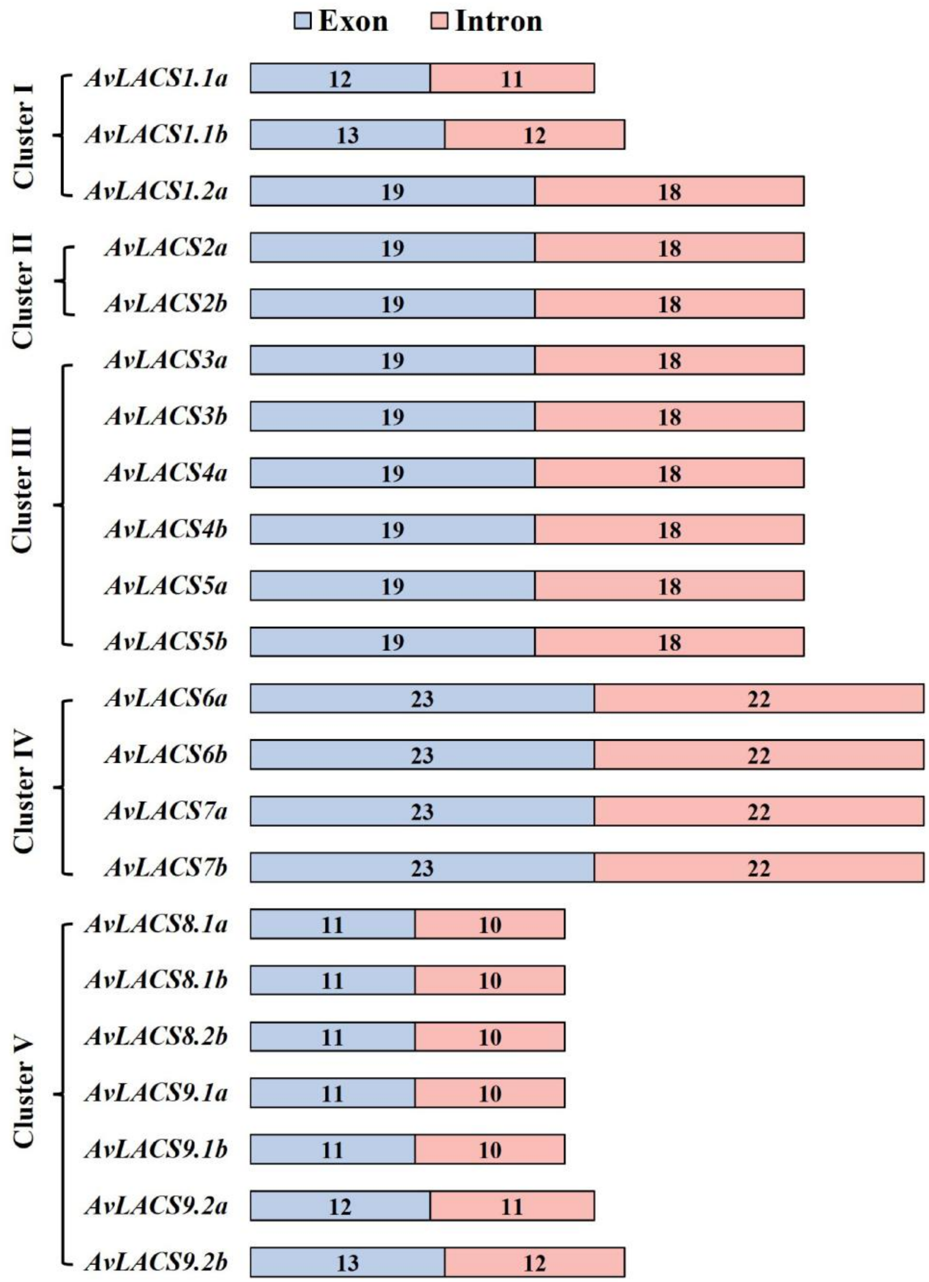


**Supplementary Figure 6**. Numbers of exon–intron pairs in the *AvLACS* gene family.

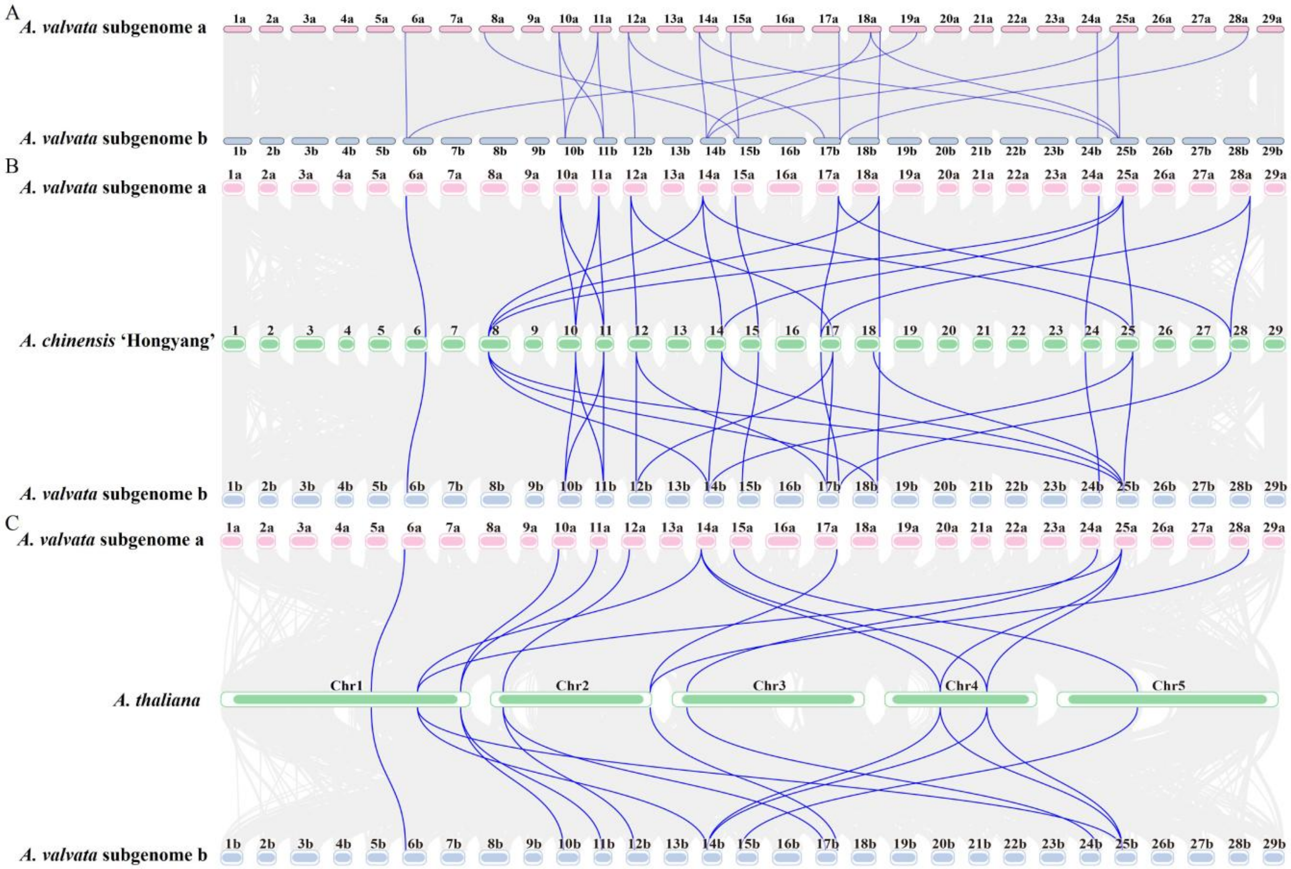


**Supplementary Figure 7**. Multiple collinearity analysis between *A. valvata*, *A. chinensis* 'Hongyang' and *A. thaliana*. (A) The collinearity analysis between *A. valvata* subgenome a and b. (B) The collinearity analysis between *A. valvata* subgenome and *A. chinensis* 'Hongyang'. (C) The collinearity analysis between *A. valvata* subgenome and *A. thaliana*. The grey lines in the background represent all syntenic blocks, while the blue lines highlight the *LACS* orthologous genes. The differently colored columns represent the chromosomes.
